# Toward Objective, Morphology-Based Taxonomy: A Case Study on the Malagasy *Nesomyrmex sikorai* Species Group (Hymenoptera: Formicidae)

**DOI:** 10.1371/journal.pone.0152454

**Published:** 2016-04-20

**Authors:** Sándor Csősz, Brian L. Fisher

**Affiliations:** Department of Entomology, California Academy of Sciences, 55 Music Concourse Drive, San Francisco, CA 94118, United States of America; Universidade de São paulo, BRAZIL

## Abstract

Madagascar is one of the world’s greatest biodiversity hotspots, meriting special attention from biodiversity scientists. It is an excellent testing ground for novel techniques in taxonomy that aim to increase classification objectivity and yield greater taxonomic resolving power. Here we reveal the diversity of a unique and largely unexplored fragment of the Malagasy ant fauna using an advanced combination of exploratory analyses on quantitative morphological data allowing for increased objectivity in taxonomic workflow. The diversity of the *Nesomyrmex sikorai* species-group was assessed via hypothesis-free *nest-centroid-clustering* combined with *recursive partitioning* to estimate the number of morphological clusters and determine the most probable boundaries between them. This combination of methods provides a highly automated and objective species delineation protocol based on continuous morphometric data. Delimitations of clusters recognized by these exploratory analyses were tested via confirmatory Linear Discriminant Analysis (LDA) and Multivariate Ratio Analysis (MRA). The final species hypotheses are corroborated by many qualitative characters, and the recognized species exhibit different spatial distributions and occupy different ecological regions. We describe and redescribe eight morphologically distinct species including six new species: *Nesomyrmex excelsior* **sp. n.**, *N*. *modestus* **sp. n.**, *N*. *reticulatus* **sp. n.**, *N*. *retusispinosus* (Forel, 1892), *N*. *rugosus* **sp. n.**, *N*. *sikorai* (Emery, 1896), *N*. *striatus* **sp. n.**, and *N*. *tamatavensis* **sp. n.** An identification key for their worker castes using morphometric data is provided.

## Introduction

The main objective of taxonomy is to document the diversity of flora and fauna. It provides fundamental information for endeavors dealing with biodiversity by addressing a crucial question: “how many species are there?” Unfortunately, the rapidly accelerating rate of biodiversity loss we face today [1, 2] poses new challenges to systematic research.

In recent years a number of promising new algorithmic approaches has been developed and introduced in insect taxonomy for the purpose of recognizing complex patterns in continuous morphometric data [3–7]. These methods help to reveal morphological diversity based on multivariate morphometric analyses [8, 9], but are prone to bias as the number of clusters is still user-defined.

In this paper the diversity of the Malagasy *Nesomyrmex sikorai* group is inferred via a highly automated protocol involving a fusion of two algorithms, Nest Centroid clustering [7] and Partitioning Algorithm based on Recursive Thresholding [10]. The *Nesomyrmex sikorai* species group is defined and diagnosed by Csősz & Fisher [11] as follows: anterodorsal spines on petiolar node absent; petiolar node long and narrow in dorsal view, sides are nearly parallel; propodeal spines moderately long, always present, mesopropodeal depression conspicuous, deep; postocular distance vs. petiole width = 1.415 [min = 1.198, max = 1.676].

Benefits of the combined application of Nest Centroid clustering (NC clustering) and Partitioning Algorithm based on Recursive Thresholding (PART) was tested and proved efficient in species delimitation in *Nesomyrmex* [11]. The NC clustering searches for discontinuity in morphometric data by sorting all similar cases into clusters in a two-step procedure. The first step reduces dimensionality of the data and computes linear discriminants (LD) for all samples as coordinates in a morphospace. Removal of collinearity between variables improves the performance of the machine learning model. The second step calculates pairwise distances between samples using LD scores as input, and displays the distance matrix in a dendrogram. The latter step is responsible for better visualization of the data. This technique has proved efficient at pattern recognition within large and complex datasets [12–14], but the number of clusters is still subjectively defined.

The PART method offers an excellent opportunity to determine the number of clusters based on statistic thresholds. It uncovers distinct subgroups in the groups identified via recursive application of the Gap statistic algorithm [15] using the LD scores generated by the NC clustering as input. A crucial feature of the algorithm is the introduction of tentative splits of clusters to isolate outliers that might otherwise halt the recursion prematurely [10].

Multivariate evaluation of morphological data has revealed that the *N*. *sikorai* species-group comprises eight well-outlined clusters in the Malagasy zoogeographical region, all representing species; of these, six are new to science. The eight species outlined are described or redefined here based on worker caste are as follows: *Nesomyrmex excelsior* **sp. n.**, *N*. *modestus* **sp. n.**, *N*. *reticulatus* **sp. n.**, *N*. *retusispinosus* (Forel, 1892), *N*. *rugosus* **sp. n.**, *N*. *sikorai* (Emery, 1896), *N*. *striatus* **sp. n.** and *N*. *tamatavensis* **sp. n.**. We provide a combined key using both qualitative and quantitative characters. Morphological patterns are linked to geographic map elevations of the sites where populations were collected and are also provided as predictor variables.

Our research has also revealed that the dimensionality reduction feature and graphical display of the NC clustering aligned with PART that automatically assigns samples into clusters allows rapid and objective decision-making in morphometry-based alpha taxonomy. Fusion of PART with NC clustering helps to readily infer species boundaries, thereby greatly reduces the need for subjective interpretation.

## Material and Methods

Ant samples used in this study comply with the regulations for export and exchange of research samples outlined in the Convention of Biology Diversity and the Convention on International Trade in Endangered Species of Wild Fauna and Flora. For field work conducted in Madagascar, permits to research, collect and export ants were obtained from the Ministry of Environment and Forest as part of an ongoing collaboration between the California Academy of Sciences and the Ministry of Environment and Forest, Madagascar National Parks and Parc Botanique et Zoologique de Tsimbazaza. Authorization for export was provided by the Director of Natural Resources.

In the present study, 22 continuous morphometric traits were recorded in 227 worker individuals belonging to 180 nest samples collected in the Malagasy region and deposited in the following institutions, abbreviations after [16]: CASC (California Academy of Sciences, San Francisco, California, U.S.A.), MCZ (Museum of Comparative Zoology, Cambridge, Massachusetts, U.S.A.), MHNG (Muséum d’Histoire Naturelle, Geneva, Switzerland). The full list of material morphometrically examined in this revision is listed in S1 Table with unique specimen identifiers (e.g. CASENT0374465). Designation of type material with detailed label information is given in the *type material investigated* sections for each taxon.

All images and specimens used in this study are available online on AntWeb (http://www.antweb.org). Images are linked to their specimens via the unique specimen code affixed to each pin (CASENT0374465). Online specimen identifiers follow this format: http://www.antweb.org/specimen/CASENT0374465.

Digital color montage images were created using a JVC KY-F75 digital camera and Syncroscopy Auto-Montage software (version 5.0), or a Leica DFC 425 camera in combination with the Leica Application Suite software (version 3.8). Distribution maps were generated in R [17] using package phytools [18].

The measurements were taken with a Leica MZ 12.5 stereomicroscope equipped with an ocular micrometer at a magnification of 100×. Measurements and indices are presented as arithmetic means with minimum and maximum values in parentheses. Body size dimensions are expressed in μm. Due to the abundance of worker individuals in contrast to the limited number of queen and male specimens available, the present revision is based on worker caste only. Worker-based revision is further facilitated by the fact that the name-bearing type specimens of the vast majority of existing ant taxa belong to the worker caste. All measurements were made by the first author. The following morphometric traits are defined in earlier works [9, 11]. Morphological variation, stability and discriminatory power of these characteristics have been shown to be suitable for the purpose of separating morphospecies of the genera *Temnothorax* [9] and *Nesomyrmex* [11]. For the definition of morphometric characters, earlier protocols [11] were considered. The repeatability of morphometric traits was tested. All variables have been measured twice for 14 randomly chosen ant specimens [19], the average measure of intraclass correlation coefficient were calculated [20]. Results of the repeatability tests are given in parentheses [R=] for each morphometric trait after character definitions with up to three decimal places. Explanations and abbreviations for measured characters are as follows:

### Nomenclature Acts

The electronic edition of this article conforms to the requirements of the amended International Code of Zoological Nomenclature, and hence the new names contained herein are available under that Code from the electronic edition of this article. This published work and the nomenclatural acts it contains have been registered in ZooBank, the online registration system for the ICZN. The ZooBank LSIDs (Life Science Identifiers) can be resolved and the associated information viewed through any standard web browser by appending the LSID to the prefix “http://zoobank.org/”. The LSID for this publication is: lsid:zoobank.org:pub:0779FA9E-22C2-4C12-A3C6-6FCA61887E70. The electronic edition of this work was published in a journal with an ISSN, and has been archived and is available from the following digital repositories: PubMed Central, LOCKSS.

### Protocol of character recording

**Fig 1:**
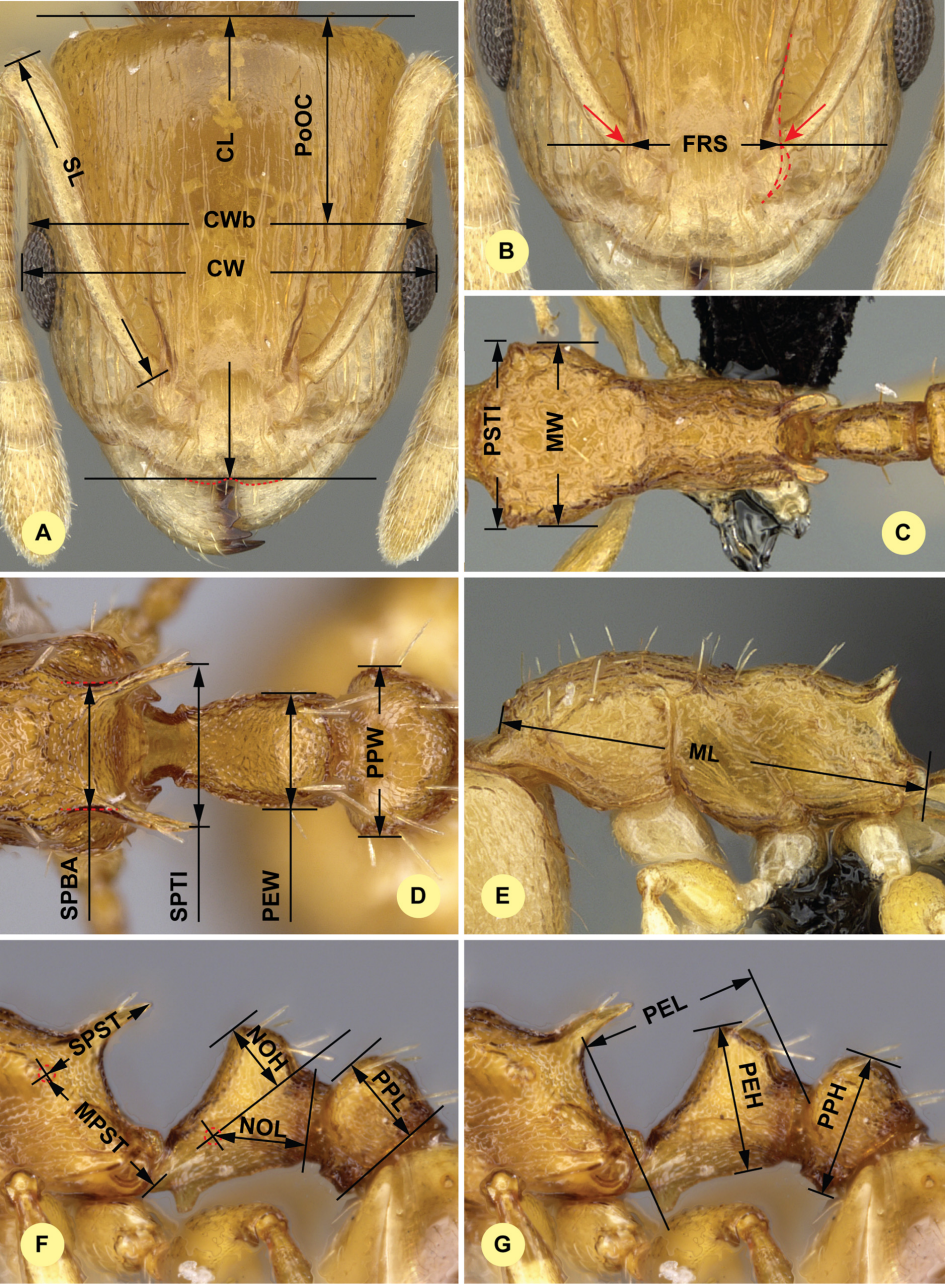
Definition of morphological characters of workers of *Nesomyrmex sikorai species*-complex. (A) Head in dorsal view with measurement lines for CL, CW, CWb, PoOC and SL. (B) Frontal region of the head dorsum with measurement lines for FRS (red accessory lines and arrows identify the torular lamella). (C) Dorsal view of mesosoma with measurement lines for PSTI and MW. (E) Dorsal view of propodeum, petiole and postpetiole with measurement lines for SPBA, SPTI, PEW and PPW. (F) Lateral view of mesosoma with measurement lines for ML. (6) Lateral view of propodeum, petiole and postpetiole with measurement lines for MPST, NOH, NOL, PPL and SPST. (G) Lateral view of propodeum, petiole and postpetiole with measurement lines for PEH, PEL and PPH.

CL: Maximum cephalic length in median line. The head must be carefully tilted to the position providing the true maximum. Excavations of hind vertex and/or clypeus reduce CL, [R = 0.998] (Fig 1A).

CW: Maximum width of the head including compound eyes, [R = 0.999] (Fig 1A).

CWb: Maximum width of head capsule without the compound eyes. Measured just posterior to the eyes, [R = 0.998] (Fig 1A).

PoOC: Postocular distance. Use a cross-scaled ocular micrometer and adjust the head to the measuring position of CL. Caudal measuring point: median occipital margin; anterior measuring point: median head at the level of the posterior eye margin, [R = 0.997] (Fig 1A).

SL: Scape length. Maximum straight line scape length excluding the basal neck and the articular condyle, [R = 0.998] (Fig 1A).

CS: Absolute cephalic size. The arithmetic mean of CL and CWb.

EL: Maximum diameter of the compound eye, [R = 0.929].

FRS: Frontal carina distance. Distance of the frontal carinae immediately caudal of the posterior intersection points between frontal carinae and torular lamellae. If these dorsal lamellae do not laterally surpass the frontal carinae, the deepest point of scape corner pits may be taken as the reference line. These pits take up the inner corner of the scape base when the scape is directed fully caudally and produces a dark, triangular shadow in the lateral frontal lobes immediately posterior to the dorsal lamellae of the scape joint capsule, [R = 0.982] (Fig 1B).

MW: Mesosoma width. In workers MW is defined as the longest width of the pronotum in dorsal view excluding the pronotal spines, [R = 0.998] (Fig 1C).

PSTI: Apical distance of pronotal spines in dorsal view; if spine tips are rounded or thick take the centers of spine tips as reference points, [R = 0.994] (Fig 1C).

PEW: Maximum width of petiole in dorsal view. Nodal spines are not considered, [R = 0.996] (Fig 1D).

PPW: Postpetiole width. Maximum width of postpetiole in dorsal view, [R = 0.994] (Fig 1D).

SPBA: Minimum propodeal spine distance. The smallest distance of the lateral margins of the propodeal spines at their base. This should be measured in antero-dorsal view, since the wider parts of the ventral propodeum do not interfere with the measurement in this position. If the lateral margins of propodeal spines diverge continuously from the tip to the base, a smallest distance at base is not defined. In this case, SPBA is measured at the level of the bottom of the interspinal meniscus, [R = 0.993] (Fig 1D).

SPTI: Apical propodeal spine distance. The distance of propodeal spine tips in dorsal view; if spine tips are rounded or truncated, the centers of spine tips are taken as reference points, [R = 0.994] (Fig 1D).

ML (Weber’s length): Mesosoma length from caudalmost point of propodeal lobe to transition point between anterior pronotal slope and anterior pronotal shield (preferentially measured in lateral view; if the transition point is not well defined, use dorsal view and take the center of the dark-shaded borderline between pronotal slope and pronotal shield as anterior reference point), [R = 0.998] (Fig 1E).

MPST: Maximum distance from the center of the propodeal spiracle to the posteroventral corner of the ventrolateral margin of the metapleuron, [R = 0.989] (Fig 1F).

NOH: Maximum height of the petiolar node, measured in lateral view from the uppermost point of the petiolar node perpendicular to a reference line set from the petiolar spiracle to the imaginary midpoint of the transition between the dorso-caudal slope and dorsal profile of caudal cylinder of the petiole, [R = 0.958] (Fig 1F).

NOL: Length of the petiolar node. Measured in lateral view from the center of the petiolar spiracle to the dorso-caudal corner of caudal cylinder. Do not erroneously take as the reference point the dorso-caudal corner of the helcium, which is sometimes visible, [R = 0.981] (Fig 1F).

PPL: Postpetiole length. The longest anatomical line that is perpendicular to the posterior margin of the postpetiole and is between the posterior postpetiolar margin and the anterior postpetiolar margin, [R = 0.975] (Fig 1F).

SPST: Propodeal spine length. Distance between the center of the propodeal spiracle and spine tip. The spiracle center refers to the midpoint defined by the outer cuticular ring but not to the center of the actual spiracle opening, which may be positioned eccentrically, [R = 0.994] (Fig 1F).

PEH: Maximum petiole height. The longest distance measured from the ventral petiolar profile at node level (perpendicular to the chord length of the petiolar sternum) to the distalmost point of the dorsal profile of the petiolar node, [R = 0.989] (Fig 1G).

PEL: Diagonal petiolar length in lateral view; measured from anterior corner of subpetiolar process to dorso-caudal corner of caudal cylinder, [R = 0.991] (Fig 1G).

PPH: Maximum height of the postpetiole in lateral view measured perpendicularly to a line defined by the linear section of the segment border between postpetiolar tergite and sternite, [R = 0.991] (Fig 1G).

Taxonomic nomenclature, OTU concepts, and natural language (NL) phenotypes were compiled in mx (http://purl.org/NET/mx-database). Taxonomic history and descriptions of taxonomic treatments were rendered from this software. Hymenoptera-specific terminology of morphological statements used in descriptions, the identification key, and diagnoses are mapped to classes in phenotype-relevant ontologies (Hymenoptera Anatomy Ontology (HAO) [21] via a URI table (S2 Table); see [22, 23] for more information about this approach. A character matrix for the taxa treated in this revisionary work, extracted from the MX database, is given in S1 Appendix.

In verbal descriptions of taxa based on external morphological traits, recent taxonomic papers [11], [12] were considered. Definitions of surface sculpturing are linked to Harris [24]. Body size is given in μm, and the means of morphometric ratios as well as minimum and maximum values are given in parentheses with up to three digits. Estimated inclination of pilosity and cuticular spines is given in degrees. Definitions of species-groups as well as descriptions of species are presented in alphabetic order.

### Statistical framework—hypothesis formation and testing

#### Data preparation and cleaning

Nest-centroid clustering (NC-clustering), and linear discriminant analysis (LDA) do not require special data preparation (e.g. standardization), hence raw data were applied for each of the statistical analyses. Data, however, are standardized (i.e., centered and scaled) for the multivariate ratio analysis (MRA) to prevent variables that are larger from dominating the analysis [5]. Variables are tested via matrix scatterplots and Pearson product-moment correlation coefficients for error variance. The lack of positive within-class correlation between different traits may indicate low repeatability of a character, or may represent a morphological artifact [8]. Each trait shown to have a strong linear correlation to other traits, therefore, was involved in multivariate analyses. Raw data in μm is given in S3 Table.

#### Generating prior species hypotheses by combined application of NC clustering and method PART

This method searches for discontinuities in continuous morphometric data and sorts all similar cases into the same cluster in a two-step procedure. The first step reduces dimensionality in data by a cumulative linear discriminant analysis (LDA) using nest samples (i.e., individuals collected from the same nest are assumed genetically closely related, often sisters) as groups [7]. The second step calculates pairwise distances between samples using LD scores as input. The distance matrix is displayed as a dendrogram. The NC-clustering was done via packages *cluster* [25] and *MASS* [26].

The ideal number of clusters was determined by Partitioning Algorithm based on Recursive Thresholding via the package clusterGenomics [27] using the function (part), which also assigns observations (i.e. specimens, or samples) into partitions. The method estimates the number of clusters in a data based on recursive application of the Gap statistic [28] and is able to discover both top-level clusters as well as sub-clusters nested within the main clusters. If more than one cluster is returned by the Gap statistic, it is re-optimized on each subset of cases corresponding to a cluster until a stopping threshold is reached or the subset under evaluation has less than 2*minSize cases [10]. Method two clustering methods are used to determine optimal number of clusters "hclust" and "kmeans" with 500 bootstrap iterations. The results of PART is mapped on the dendrogram by colored bars via function ‘mark.dendrogram’ found in [29]. This protocol has been introduced by Csősz & Fisher [30]. The script written in R is available in S2 Appendix.

#### Arriving at final species hypothesis using confirmatory Linear Discriminant Analysis (LDA) and LDA ratio extractor

To provide increased reliability of species delimitation, hypotheses on clusters and classification of cases via exploratory processes were confirmed by LDA Leave-one-out cross-validation (LOOCV). Classification hypotheses were imposed for all samples congruently classified by partitioning methods while wild-card settings (i.e. no prior hypothesis imposed on its classification) were given to samples that were incongruently classified by the two methods. To extract the best ratios for the easiest species separation in the key and diagnoses we applied multivariate ratio analysis (MRA), a modern statistical method based on principal component analysis (PCA) and linear discriminant analysis (LDA) [5].

## Results and Discussion

**Fig 2:**
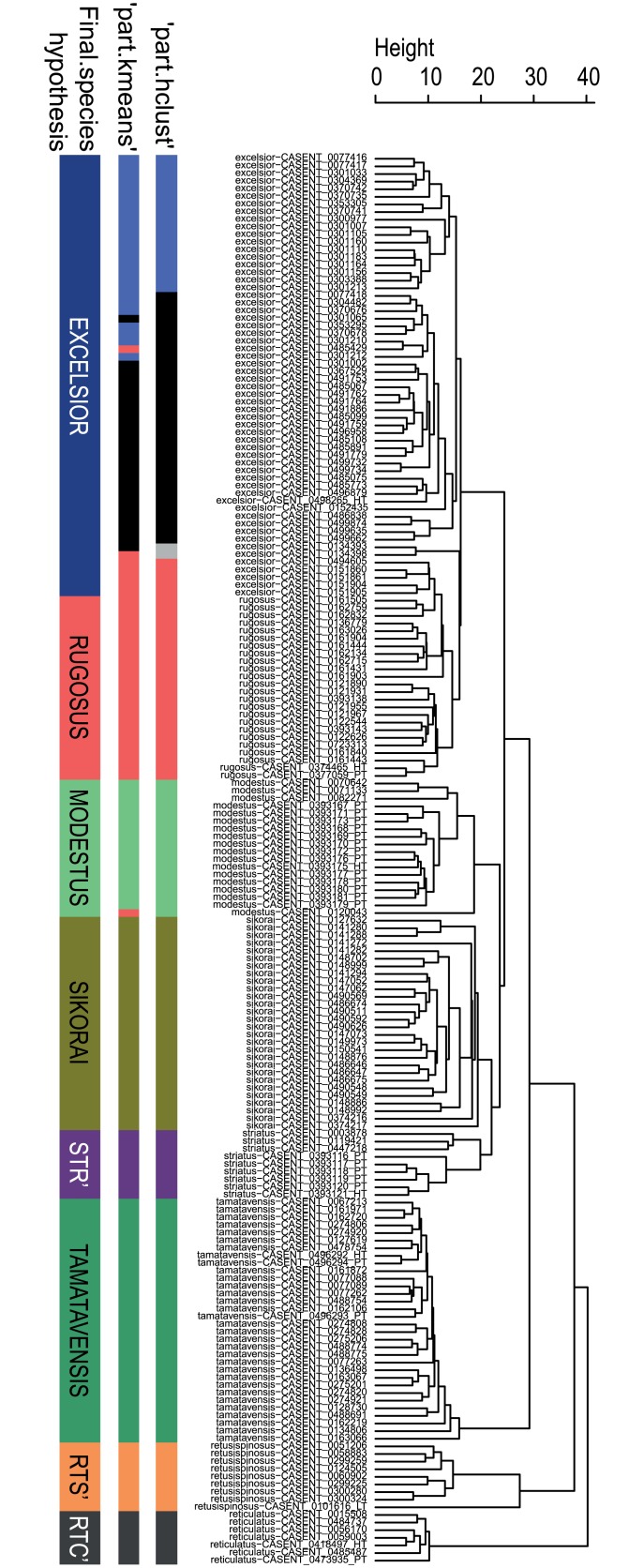
Dendrogram solution for *Nesomyrmex sikorai* species group. Sample information in the dendrogram follows the given format: final species hypothesis confirmed by cross-validation LDA is followed by CASENT number separated by a hyphen. Final species hypothesis bar shows classification of samples after confirmation by cross-validated LDA. Different colors represent species. *Nesomyrmex excelsior* sp. n.: dark blue, *N*. *modestus* sp. n.: light green, *N*. *reticulatus* sp. n. (RTC’) grey, *N*. *retusispinosus* (RTS’): orange, *N*. *rugosus* sp. n.: red, *N*. *sikorai*: khaki, *N*. *striatus* sp. n. (STR’): lilac, *N*. *tamatavensis* sp. n.: dark green. Prior species hypothesis was generated by applying the partitioning method PART using two clustering methods, hclust (‘part-hclust’) and kmeans (‘part-kmeans’). Color code is the same as above, subclusters differently returned by two clustering methods within *N*. *excelsior* (dark blue in final species hypothesis) are split into light blue and black. Due to this discrepancy, the lack of other biological evidence, and weak support of LDA, light blue and black clusters are lumped into one group: *N*. *excelsior*. For further explanation, see the text.

Both clustering algorithms ‘hclust’ and ‘kmeans’ using function ‘part’ returned 9 clusters. The pattern recognized by these partitioning algorithms can be fitted on the hierarchical structure seen on the dendrogram generated by NC-clustering (Fig 2).

The cross-validated (LOOCV) LDA confirmed separation of eight of the nine clusters recognized by the partitioning methods. Separation of the two slightly conflicting clusters within “excelsior” (shown in black and blue in Fig 2) returned by both clustering methods is not confirmed convincingly by the LOOCV-LDA, hence remain lumped in “excelsior.” Four additional samples placed into “rugosus” by the PART method have also been reclassified as “excelsior” by cross-validation LDA; the new classification is also supported by other biological evidence such as geographic and elevational distributions of populations.

The eight-species hypothesis is confirmed by cross-validation LDA. The overall classification success is 99.12%, and each cluster but “excelsior” is separated with 100% classification success, while the latter attains 97.6% success.

**Fig 3:**
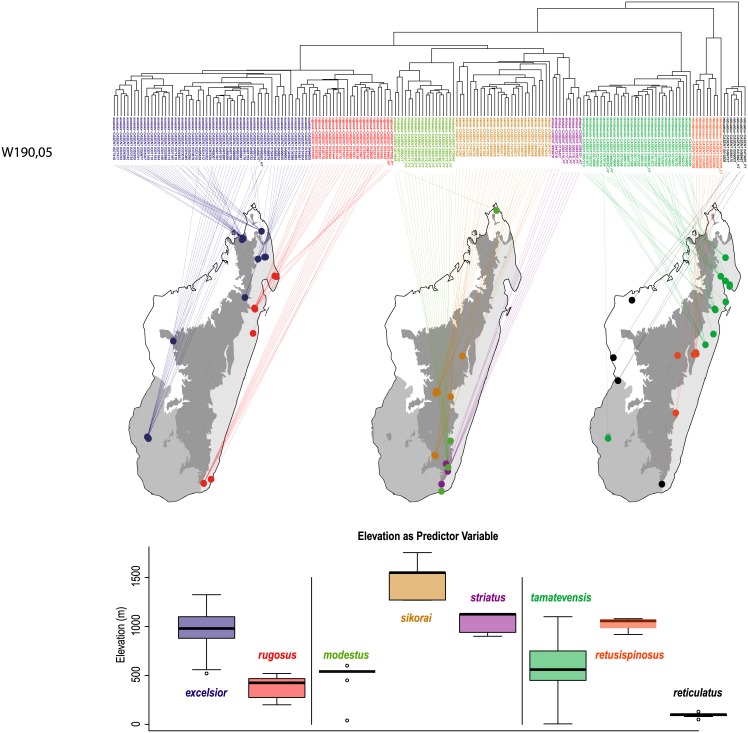
Dendrogram to geographic map. Species distributions are mapped over the outlines of four simplified ecoregion zones of Madagascar (Schatz 2000; following Cornet 1974): eastern rainforest (light gray), central montane forest (dark gray), western dry forest (white), and southwest desert spiny bush thicket (medium gray). Color codes for species are as follows: *Nesomyrmex excelsior* sp. n.: dark blue, *N*. *modestus* sp. n.: light green, *N*. *reticulatus* sp. n. (RTC’) grey, *N*. *retusispinosus* (RTS’): orange, *N*. *rugosus* sp. n.: red, *N*. *sikorai*: khaki, *N*. *striatus* sp. n. (STR’): lilac, *N*. *tamatavensis* sp. n.: dark green.

The phenetically-distinguishable clusters represent eight morphologically diagnosable OTUs that differ in many qualitative characters (e.g. shape of propodeal spines, surface sculpturing, color characteristics etc.), hence the eight clusters solution is accepted as the final species hypothesis. These species are also known to occupy different niches, and exhibit different spatial distributions. The geographic distribution of each morphospecies corresponds to the known major areas of endemism in Madagascar [31, 32] and can be characterized by one of the simplified bioclimatic zones of Madagascar [33], (after Cornet [34]): eastern rainforest, central montane forest, western dry forest, and southwest desert spiny bush thicket (Fig 3).

The eight species described here are as follows in alphabetic order: *Nesomyrmex excelsior* sp. n., *N*. *modestus* sp. n., *N*. *reticulatus* sp. n., *N*. *retusispinosus* (Forel, 1892), *N*. *rugosus* sp. n., *N*. *sikorai* (Emery, 1896), *N*. *striatus* sp. n., *N*. *tamatavensis* sp. n.

**Fig 4:**
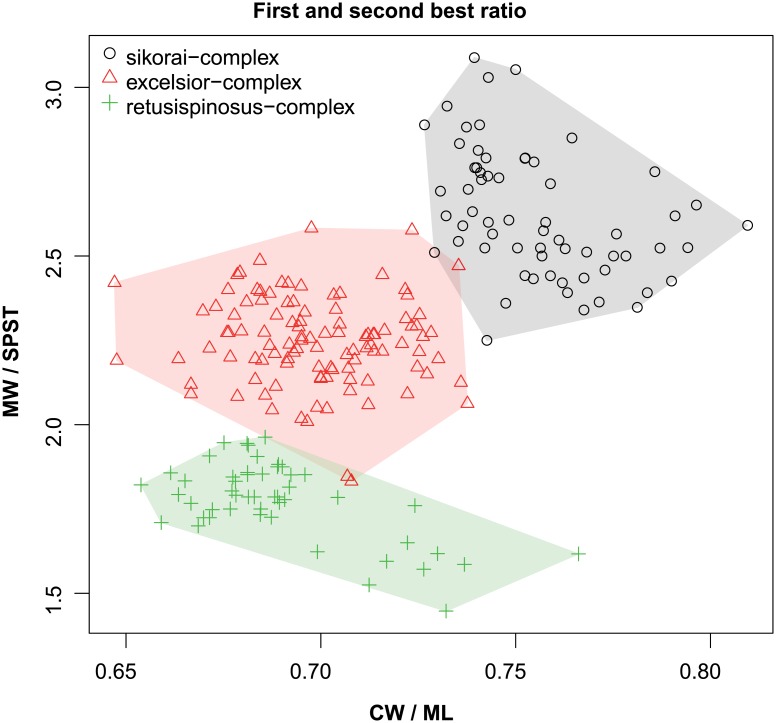
Scatterplots of the two most discriminating ratios for workers of *Nesomyrmex sikorai* complex species complexes. Color codes: *Nesomyrmex sikorai* complex (grey circles), *N*. *excelsior* complex (red triangles) and *N*. *retusispinosus* complex (green crosses).

**Fig 5:**
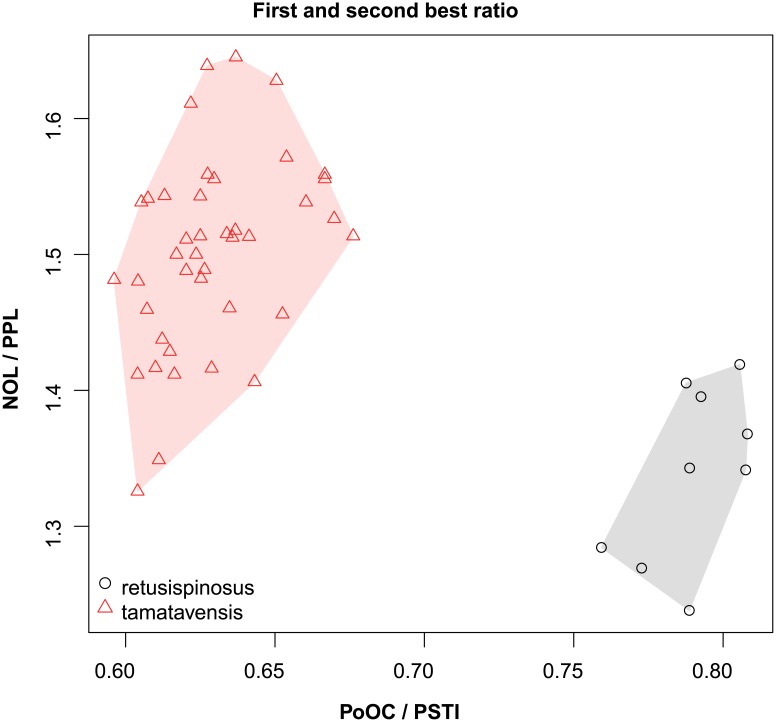
Scatterplots of the two most discriminating ratios between workers of *Nesomyrmex retusispinosus* and *N*. *tamatavensis*. Color codes: *Nesomyrmex retusispinosus* (grey circles) and *N*. *tamatavensis* (red triangles).

**Fig 6:**
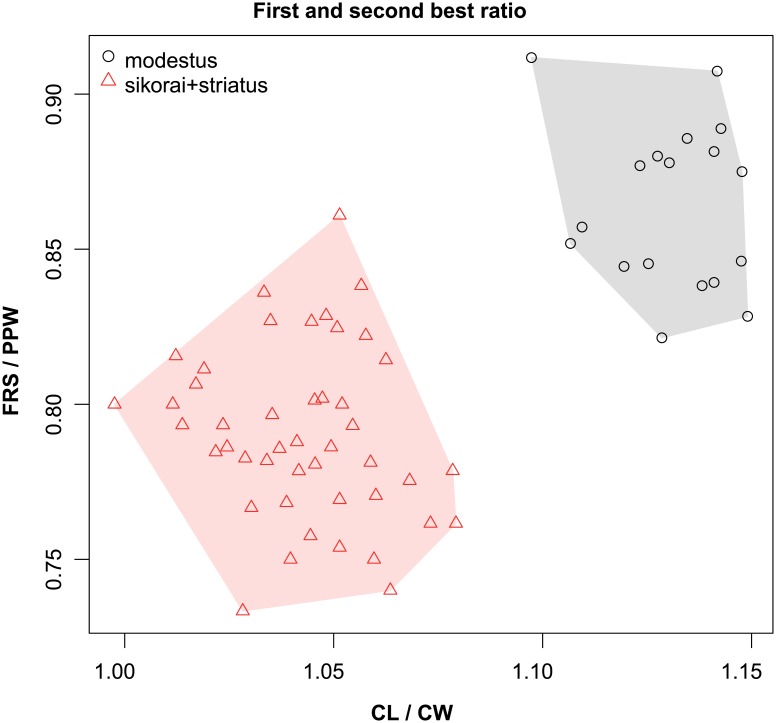
Scatterplots of the two most discriminating ratios between workers of *Nesomyrmex modestus* and the combined pool of *N*. *sikorai* and *N*. *striatus*. Color codes: *Nesomyrmex modestus* (grey circles), *N*. *sikorai* and *N*. *striatus* together (red triangles).

**Fig 7:**
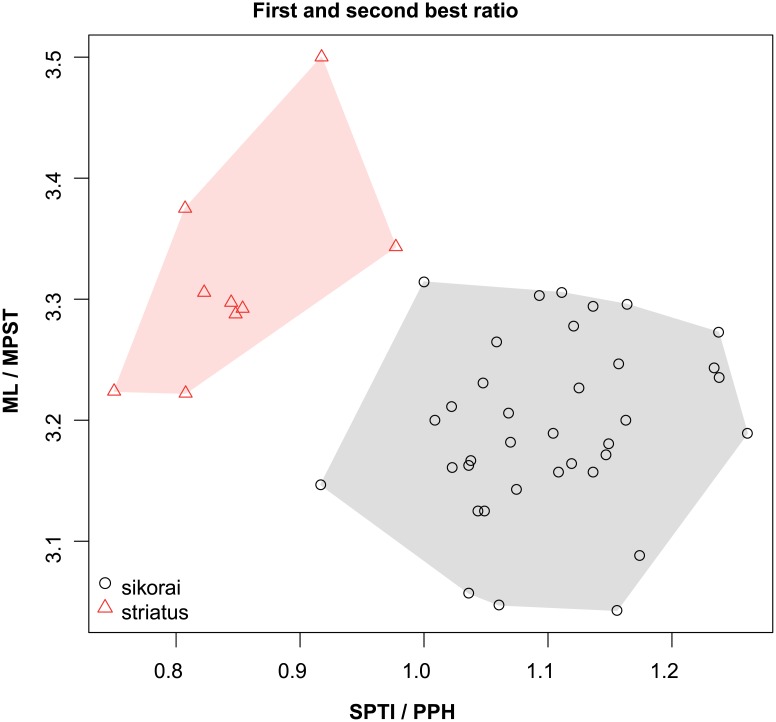
Scatterplots of the two most discriminating ratios between workers of *Nesomyrmex sikorai* and *N*. *striatus*. Color codes: *Nesomyrmex sikorai* (grey circles), *N*. *striatus* (red triangles).

**Fig 8:**
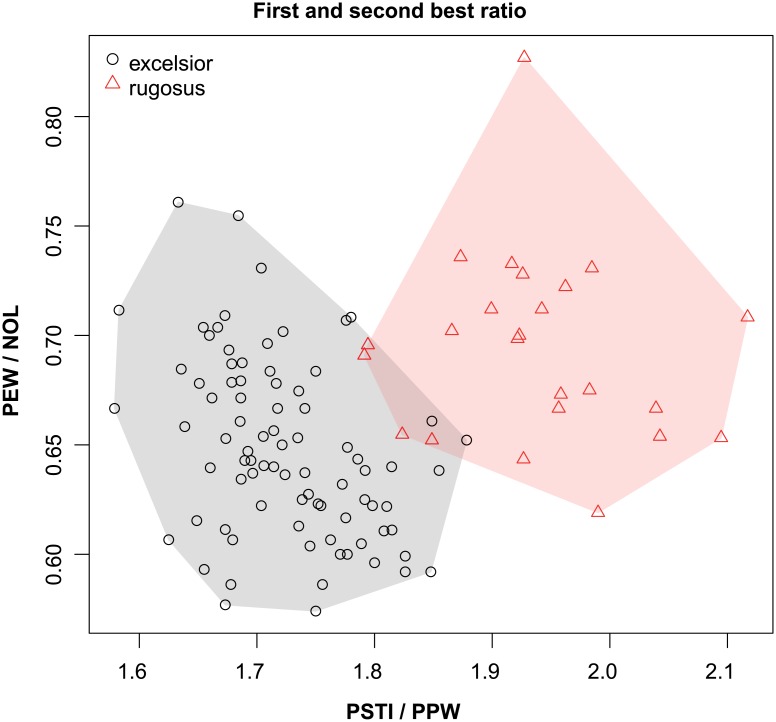
Scatterplots of the two most discriminating ratios between workers of *Nesomyrmex excelsior* and *N*. *rugosus*. Color codes: *Nesomyrmex excelsior* (grey circles), *N*. *rugosus* (red triangles).

These species are grouped into three species complexes by morphological similarity. The *retusispinosus* complex consists of two species: *N*. *retusispinosus* (Forel, 1892) and *N*. *tamatavensis* sp. n.; the *excelsior* complex includs two new species: *Nesomyrmex excelsior* sp. n. and *N*. *rugosus* sp. n.; the *sikorai* complex contains three species: *N*. *modestus* sp. n., *N*. *sikorai* (Emery, 1896), *N*. *striatus* sp. n.; while *N*. *reticulatus* sp. n. forms a complex of its own in the Malagasy zoogeographical region. Separation of species as well as complexes is also confirmed by Multivariate Ratio Analyses. Species complexes can be separated based on two morphometric ratios; MW/SPST yields a separation between the *retusispinosus* complex and others and CW/ML helps discriminate between the *excelsior* and *sikorai* complexes (Fig 4). *Nesomyrmex retusispinosus* and *N*. *tamatavensis* sp. n. can be easily separated making use of a single ratio PoOC/PSTI (Fig 5); CL/CW helps to separate *N*. *modestus* sp. n. from *N*. *sikorai* (Emery, 1896) and *N*. *striatus* sp. n. (Fig 6); while the two latter species form two non-overlapping clusters by using a combination of the two ratios shown in Fig 7. Two species placed in the *excelsior* complex, *N*. *excelsior* sp. n. and *N*. *rugosus* sp. n., form slightly overlapping clusters in MRA plot (Fig 8). The best ratio (PSTI/PPW) tells the two species apart with a 6.5% error rate. Morphometric data for species calculated on individuals are given in Table 1.

**Table 1:** Morphometric data of species calculated on individuals.

species:	N. excelsior sp.n.	N. modestus sp.n.	N. reticulatus sp.n.	N. retusispinosus	N. rugosus sp.n.	N. sikorai	N. striatus sp.n.	N. tamatavensis sp.n.
*nr*. *of individulals*:	*(n = 83)*	*(n = 18)*	*(n = 7)*	*(n = 9)*	*(n = 24)*	*(n = 36)*	*(n = 9)*	*(n = 41)*
**CS (μm)**	**736±34.3**	**796±27.6**	**511±25.7**	**812±50**	**732±35.9**	**822±34**	**842±41.3**	**699±29.3**
	[671, 839]	[743, 834]	[472, 545]	[731, 921]	[650, 781]	[748, 890]	[775, 889]	[634, 750]
**PoOC/CL**	**0.458±0.008**	**0.453±0.008**	**0.462±0.011**	**0.483±0.010**	**0.473±0.011**	**0.488±0.016**	**0.460±0.010**	**0.433±0.009**
	[0.437, 0.477]	[0.438, 0.466]	[0.449, 0.476]	[0.464, 0.498]	[0.450, 0.493]	[0.451, 0.511]	[0.441, 0.472]	[0.411, 0.449]
**CL/CW**	**1.169±0.030**	**1.131±0.015**	**1.105±0.026**	**1.089±0.037**	**1.155±0.020**	**1.041±0.019**	**1.048±0.016**	**1.107±0.016**
	[1.108, 1.245]	[1.097, 1.149]	[1.080, 1.146]	[1.002, 1.127]	[1.112, 1.191]	[0.998, 1.079]	[1.022, 1.073]	[1.079, 1.135]
**CL/CWb**	**1.271±0.033**	**1.216±0.02**	**1.151±0.019**	**1.17±0.048**	**1.237±0.024**	**1.169±0.021**	**1.155±0.025**	**1.155±0.017**
	[1.209, 1.382]	[1.179, 1.256]	[1.125, 1.180]	[1.07, 1.233]	[1.189, 1.277]	[1.126, 1.226]	[1.117, 1.192]	[1.119, 1.182]
**FRS/CS**	**0.291±0.007**	**0.295±0.006**	**0.320±0.009**	**0.294±0.007**	**0.289±0.006**	**0.292±0.009**	**0.288±0.004**	**0.329±0.007**
	[0.278, 0.312]	[0.287, 0.307]	[0.310, 0.335]	[0.285, 0.302]	[0.277, 0.300]	[0.276, 0.312]	[0.282, 0.294]	[0.318, 0.347]
**SL/CS**	**0.822±0.019**	**0.771±0.02**	**0.704±0.016**	**0.838±0.018**	**0.811±0.020**	**0.828±0.018**	**0.794±0.01**	**0.798±0.014**
	[0.786, 0.872]	[0.74, 0.808]	[0.679, 0.720]	[0.812, 0.862]	[0.784, 0.852]	[0.78, 0.862]	[0.779, 0.813]	[0.773, 0.832]
**EL/CS**	**0.230±0.008**	**0.234±0.008**	**0.241±0.011**	**0.237±0.008**	**0.228±0.005**	**0.212±0.007**	**0.219±0.008**	**0.258±0.008**
	[0.210, 0.246]	[0.217, 0.25]	[0.224, 0.255]	[0.225, 0.251]	[0.219, 0.235]	[0.201, 0.224]	[0.209, 0.232]	[0.243, 0.284]
**MW/CS**	**0.625±0.015**	**0.647±0.015**	**0.639±0.009**	**0.679±0.018**	**0.639±0.024**	**0.675±0.013**	**0.671±0.009**	**0.705±0.017**
	[0.591, 0.679]	[0.632, 0.695]	[0.624, 0.654]	[0.646, 0.7]	[0.601, 0.679]	[0.654, 0.698]	[0.659, 0.686]	[0.663, 0.736]
**PSTI/CS**	**0.611±0.015**	**0.657±0.013**	**0.629±0.015**	**0.659±0.017**	**0.648±0.023**	**0.665±0.014**	**0.666±0.013**	**0.739±0.018**
	[0.573, 0.662]	[0.636, 0.686]	[0.608, 0.650]	[0.621, 0.678]	[0.605, 0.692]	[0.636, 0.691]	[0.649, 0.686]	[0.708, 0.78]
**PEW/CS**	**0.231±0.018**	**0.223±0.007**	**0.235±0.007**	**0.24±0.014**	**0.236±0.017**	**0.269±0.02**	**0.243±0.009**	**0.243±0.014**
	[0.206, 0.267]	[0.213, 0.238]	[0.222, 0.242]	[0.219, 0.262]	[0.200, 0.278]	[0.228, 0.326]	[0.229, 0.253]	[0.208, 0.27]
**PPW/CS**	**0.354±0.015**	**0.341±0.009**	**0.370±0.016**	**0.39±0.017**	**0.335±0.014**	**0.37±0.011**	**0.362±0.007**	**0.363±0.013**
	[0.321, 0.394]	[0.326, 0.358]	[0.339, 0.386]	[0.358, 0.415]	[0.308, 0.356]	[0.348, 0.391]	[0.351, 0.373]	[0.338, 0.385]
**SPBA/CS**	**0.225±0.020**	**0.236±0.007**	**0.254±0.017**	**0.24±0.013**	**0.218±0.019**	**0.253±0.017**	**0.242±0.018**	**0.244±0.014**
	[0.183, 0.272]	[0.225, 0.248]	[0.235, 0.275]	[0.219, 0.259]	[0.182, 0.256]	[0.215, 0.287]	[0.204, 0.26]	[0.221, 0.282]
**SPTI/CS**	**0.252±0.025**	**0.251±0.012**	**0.266±0.014**	**0.322±0.013**	**0.261±0.029**	**0.299±0.019**	**0.244±0.023**	**0.303±0.02**
	[0.198, 0.310]	[0.226, 0.269]	[0.244, 0.284]	[0.293, 0.332]	[0.191, 0.310]	[0.256, 0.341]	[0.191, 0.266]	[0.273, 0.35]
**ML/CS**	**1.379±0.025**	**1.308±0.017**	**1.252±0.015**	**1.362±0.021**	**1.346±0.023**	**1.352±0.027**	**1.379±0.008**	**1.422±0.021**
	[1.326, 1.450]	[1.279, 1.344]	[1.229, 1.269]	[1.339, 1.402]	[1.295, 1.405]	[1.265, 1.412]	[1.365, 1.39]	[1.362, 1.46]
**PEL/CS**	**0.528±0.017**	**0.499±0.013**	**0.441±0.014**	**0.543±0.018**	**0.511±0.016**	**0.516±0.013**	**0.513±0.01**	**0.544±0.012**
	[0.490, 0.565]	[0.485, 0.53]	[0.424, 0.459]	[0.521, 0.575]	[0.484, 0.534]	[0.49, 0.537]	[0.503, 0.532]	[0.518, 0.576]
**NOL/CS**	**0.357±0.015**	**0.332±0.011**	**0.266±0.010**	**0.352±0.013**	**0.340±0.013**	**0.361±0.013**	**0.339±0.013**	**0.369±0.018**
	[0.326, 0.403]	[0.312, 0.358]	[0.254, 0.279]	[0.325, 0.37]	[0.320, 0.372]	[0.329, 0.388]	[0.319, 0.355]	[0.327, 0.41]
**MPST/CS**	**0.419±0.012**	**0.404±0.009**	**0.391±0.011**	**0.42±0.01**	**0.409±0.009**	**0.423±0.01**	**0.416±0.009**	**0.427±0.009**
	[0.383, 0.447]	[0.384, 0.424]	[0.372, 0.404]	[0.402, 0.434]	[0.393, 0.432]	[0.405, 0.452]	[0.394, 0.427]	[0.407, 0.444]
**PEH/CS**	**0.296±0.011**	**0.309±0.009**	**0.327±0.016**	**0.319±0.011**	**0.297±0.008**	**0.306±0.016**	**0.304±0.006**	**0.312±0.011**
	[0.271, 0.330]	[0.288, 0.322]	[0.301, 0.346]	[0.3, 0.333]	[0.284, 0.319]	[0.278, 0.346]	[0.29, 0.312]	[0.281, 0.33]
**NOH/CS**	**0.172±0.010**	**0.179±0.009**	**0.132±0.010**	**0.185±0.013**	**0.173±0.006**	**0.173±0.011**	**0.173±0.01**	**0.181±0.008**
	[0.149, 0.194]	[0.166, 0.196]	[0.122, 0.147]	[0.165, 0.205]	[0.161, 0.186]	[0.152, 0.196]	[0.155, 0.183]	[0.165, 0.194]
**PPH/CS**	**0.272±0.012**	**0.297±0.016**	**0.280±0.022**	**0.271±0.007**	**0.268±0.016**	**0.271±0.008**	**0.288±0.021**	**0.267±0.013**
	[0.245, 0.300]	[0.262, 0.311]	[0.254, 0.312]	[0.26, 0.285]	[0.239, 0.294]	[0.248, 0.287]	[0.255, 0.315]	[0.24, 0.312]
**SPST/CS**	**0.279±0.020**	**0.236±0.01**	**0.233±0.013**	**0.43±0.018**	**0.287±0.016**	**0.269±0.015**	**0.241±0.012**	**0.388±0.015**
	[0.240, 0.352]	[0.22, 0.254]	[0.220, 0.257]	[0.4, 0.459]	[0.253, 0.314]	[0.221, 0.301]	[0.219, 0.26]	[0.364, 0.43]
**PPL/CS**	**0.253±0.017**	**0.235±0.008**	**0.246±0.013**	**0.263±0.009**	**0.235±0.009**	**0.239±0.011**	**0.241±0.007**	**0.246±0.008**
	[0.200, 0.292]	[0.226, 0.252]	[0.227, 0.266]	[0.253, 0.282]	[0.214, 0.250]	[0.211, 0.27]	[0.234, 0.252]	[0.223, 0.265]

Mean of indices, ±SD are provided in the upper row, minimum and maximum values are given in parentheses in the lower row.

Separation of eight species of the nine morphologically-delineated clusters revealed by the exploratory process, i.e. the combination of NC clustering and method PART (Partitioning based on Recursive Thresholding), have been confirmed by the LDA.

Combining NC clustering and PART helps to accelerate biodiversity studies and provides an opportunity to test and compare levels of variation in quantified morphological traits. This combined approach relies on discontinuity in quantitative phenotypic variations and helps to recognize morphologically divergent lineages believed to represent OTUs reproductively isolated at a certain level. This is generally the philosophy behind morphological approaches.

Although the visual products of NC-clustering may be reminiscent of phylogenetic trees, it is important to emphasize that the results of our analysis on continuous morphometric data are not equivalent to a phylogenetic analysis. Over generations, the gene pool of a population can undergo a row of minor changes manifesting in diverging phenotypes, forming the theoretical basis of phenetic approaches. Phenetic distances, calculated from differences in quantitative morphometric traits, may often correlate with the age of lineages and the level of reproductive isolation. However, for many features important for phylogenetic inference, shared ancestry cannot be convincingly ascertained, hence genealogical patterns based on phenetic morphological approaches should be inferred only with extreme care.

Our knowledge about the morphological evolution of traits and the development of stable alternative phenotypes generated by an environmental factor is limited. However, we believe that novel, improved algorithmic approaches applied complementarily to genetic markers offer great opportunities to test hypothesis of molecular phylogeny. Steady increases in our understanding of the development and heritability of morphological traits and and their role in stabilizing selection [35] will foster further applications of quantitative morphological traits in biodiversity research.

### Synopsis of *Nesomyrmex* species treated in this revisionary work

*excelsior* Csősz & Fisher sp. n.

*modestus* Csősz & Fisher sp. n.

*reticulatus* Csősz & Fisher sp. n.

*retusispinosus* (Forel, 1892)

*rugosus* Csősz & Fisher sp. n.

*sikorai* (Emery, 1896)

*striatus* Csősz & Fisher sp. n.

*tamatavensis* Csősz & Fisher sp. n.

### Key to the species of *sikorai* group

1. Scapes very short: SL/CS < 0.73 [0.679, 0.720], petiolar node very short: NOL/CS < 0.3 [0.254, 0.279]… N. reticulatus sp. n.
  - Scapes longer: SL/CS > 0.73 [0.74, 0.872], petiolar node longer: NOH/CS 0.3 > [0.312, 0.358]… 2
2. Propodeal spines curving downward, blunt, and very long: SPST/CS > 0.36 [0.364, 0.459]… 3
  - Propodeal spines triangular, acute, and shorter: SPST/CS < 0.36 [0.219, 0.352]… 4
3. Vertex rugose, ground sculpture areolate, dull. In full-face view, compound eyes positioned forward and closer to the anterior clypeal border: PoOC/CL > 0.455 [0.464, 0.498]… *N*. *retusispinosus* (Forel, 1892)
  - Vertex smooth, main sculpture absent, ground sculpture inconspicuously areolate, shiny. In full-face view, compound eyes positioned in the middle of the longitudinal axis of head or closer to the posterior occipital border: PoOC/CL < 0.455 [0.411, 0.449]… *N*. *tamatavensis* sp. n.
4. Black species. Eyes protuberant in full-face view: CW/ML: > 0.73 [0.727, 0.810], [5–95% percentiles: 0.732, 0.791] … 5
  - Workers yellow to light brown. Eyes non-protuberant in full-face view: CW/ML: < 0.73 [0.647, 0.738], [5–95% percentiles: 0.670, 0.727] … 7
5. Main sculpture on vertex and on the dorsum of mesosoma inconspicuously areolate or absent partly, ground sculpture smooth and shiny. Eyes moderately protuberant in full-face view: CL/CW > 1.09 [1.097, 1.149] … *N*. *modestus* sp. n.
  - Main sculpture on vertex and on the dorsum of mesosoma present and coarse areolate or costate, ground sculpture inconspicuously areolate or absent. Eyes strongly protuberant in full-face view: CL/CW < 1.09 [0.998, 1.079] … 6
6. Main sculpture on vertex coarse areolate, ground sculpture inconspicuously areolate. Dorsum of petiolar node rugoso-reticulate, dull. The best morphometric ratio PSTI/SPTI ≤ 2.5 [1.966, 2.50] … *N*. *sikorai* (Emery, 1896)
  - Main sculpture on vertex longitudinal costate, ground sculpture inconspicuously areolate. Dorsum of petiolar node smooth and shiny. The best morphometric ratio PSTI/SPTI > 2.5 [2.512, 3.40] … *N*. *striatus* sp. n.
7. Postpetiole longer, anterolateral angles of mesosoma less distant: PSTI/PPW > 1.84 [1.579, 1.878], [5–95% percentiles: 1.636, 1.826]. Occurs on higher ground > 500 m. … *N*. *excelsior* sp. n.
  - Postpetiole shorter, anterolateral angles of mesosoma widely distant: PSTI/PPW 1.84 [1.791, 2.117], [5–95% percentiles: 1.794, 2.095]. Occurs on lower elevations < 500 m. … *N*. *rugosus* sp. n.

### Description of the species of *Nesomyrmex sikorai* species-group

In this section, eight species of the *N*. *sikorai* species-group are described including biogeographic information. The basic statistics of body size ratios are given in Table 1 for each species.

### *Nesomyrmex excelsior* Csősz & Fisher sp. n.

**Fig 9:**
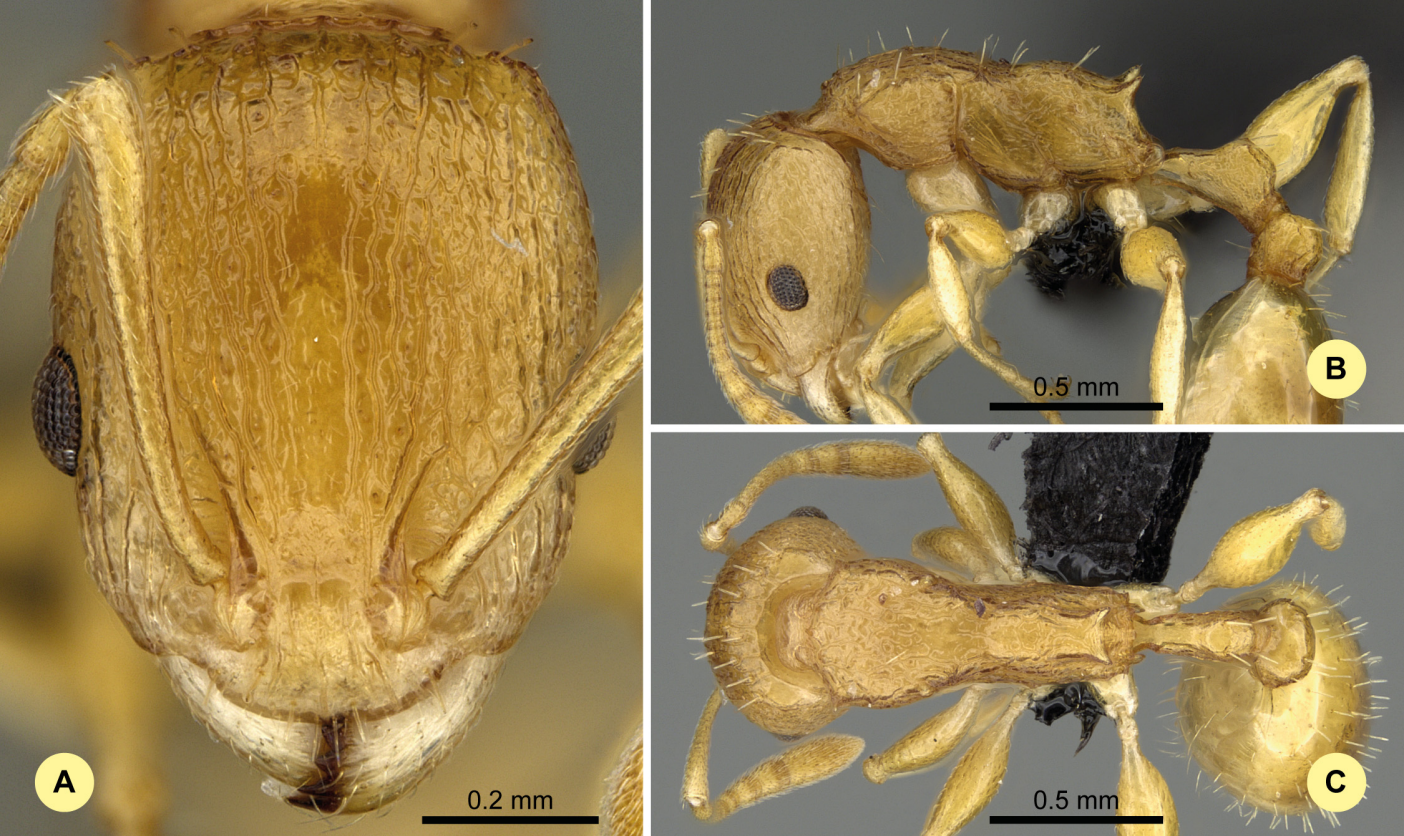
*Nesomyrmex excelsior* sp. n. holotype worker (CASENT0498265). (A) Head of the holotype worker in full-face view. (B) Lateral view of the body. (C) Dorsal view of the body.

urn:lsid:zoobank.org:act:540D306B-7EAC-4AD9-B072-842AC26F91F7

(Figs 3 and 9A–9C, Table 1.)

#### Type material investigated

Holotype worker: MADGAGASCAR: Prov. Toliara, Forêt Classée d'Analavelona, 29.2 km 343° NNW Mahaboboka, Madagascar, 1100 m, -22.675 N, 44.19 E, 18.ii.2003, collection code: BLF07826; CASENT0498265, Fisher et al., ex dead twig above ground montane rainforest (CAS, CASENT0498265);

Paratypes: 8 workers and a single gyne with the same label data with the holotype under CASENT codes: CASENT0763558, BLF07826, (2w, CAS); CASENT0498263, BLF07826, (3w, CAS); CASENT0498264, BLF07826, (3w, CAS); CASENT0498266, BLF07826, (1Q, CAS);

The list of 83 worker individuals belonging to 58 nest samples morphometrically investigated is given in S1 Table.

#### Diagnosis

See in key.

#### Description of workers

Body color: yellow; brown. Body color pattern: Body concolorous. Absolute cephalic size: 726 μm [671, 799]. Cephalic length vs. maximum width of head capsule (CL/CWb): 1.281 [1.218, 1.382]. Postocular distance vs. cephalic length (PoOc/CL): 0.457 [0.437, 0.477]. Postocular sides of cranium contour, anterior view orientation: converging posteriorly. Postocular sides of cranium contour, anterior view shape: convex. Vertex contour line in anterior view shape: straight; feebly convex. Vertex sculpture: main sculpture rugoso-reticulate, ground sculpture smooth. Gena contour line in anterior view shape: convex. Genae contour from anterior view orientation: strongly converging. Gena sculpture: rugoso-reticulate with feeble areolate ground sculpture. Concentric carinae laterally surrounding antennal foramen count: present. Eye length vs. absolute cephalic size (EL/CS): 0.233 [0.221, 0.246]. Frontal carina distance vs. absolute cephalic size (FRS/CS): 0.289 [0.278, 0.304]. Longitudinal carinae on median region of frons count: present. Smooth median region on frons count: absent. Antennomere count: 12. Scape length vs. absolute cephalic size (SL/CS): 0.822 [0.786, 0.872]. Median clypeal notch count: present. Median carina of clypeus count: absent. Spine length vs. absolute cephalic size (SPST/CS): 0.275 [0.24, 0.315]. Minimum spine distance vs. absolute cephalic size (SPBA/CS): 0.225 [0.183, 0.272]. Apical spine distance vs. absolute cephalic size (SPTI/CS): 0.252 [0.198, 0.31]. Propodeal spine shape: straight; triangular, blunt. Apical distance of pronotal spines vs. absolute cephalic size (PSTI/CS): 0.607 [0.573, 0.633]. Metanotal depression count: present. Dorsal region of mesosoma sculpture: rugose with smooth ground sculpture. Lateral region of pronotum sculpture: inconspicuously areolate ground sculpture, main sculpture rugoso-reticulate. Mesopleuron sculpture: smooth ground sculpture, superimposed by dispersed rugulae. Metapleuron sculpture: areolate ground sculpture, superimposed by dispersed rugae. Petiole width vs. absolute cephalic size (PEW/CS): 0.233 [0.206, 0.267]. Dorsal region of petiole sculpture: ground sculpture inconspicuously areolate, main sculpture rugoso-reticulate. Postpetiole width vs. absolute cephalic size (PPW/CS): 0.354 [0.321, 0.394]. Dorsal region of postpetiole sculpture: ground sculpture smooth, main sculpture dispersed rugose.

#### Etymology

The name (excelsior = higher, loftier) refers to the typically montane occurrence of this species.

#### Distribution

This species is known to occur in smaller, isolated montane rainforests between elevation of 520 m and 1325 m (mean: 1007 m) in the western and central part of Madagascar (Fig 3).

### *Nesomyrmex modestus* Csősz & Fisher sp. n.

**Fig 10:**
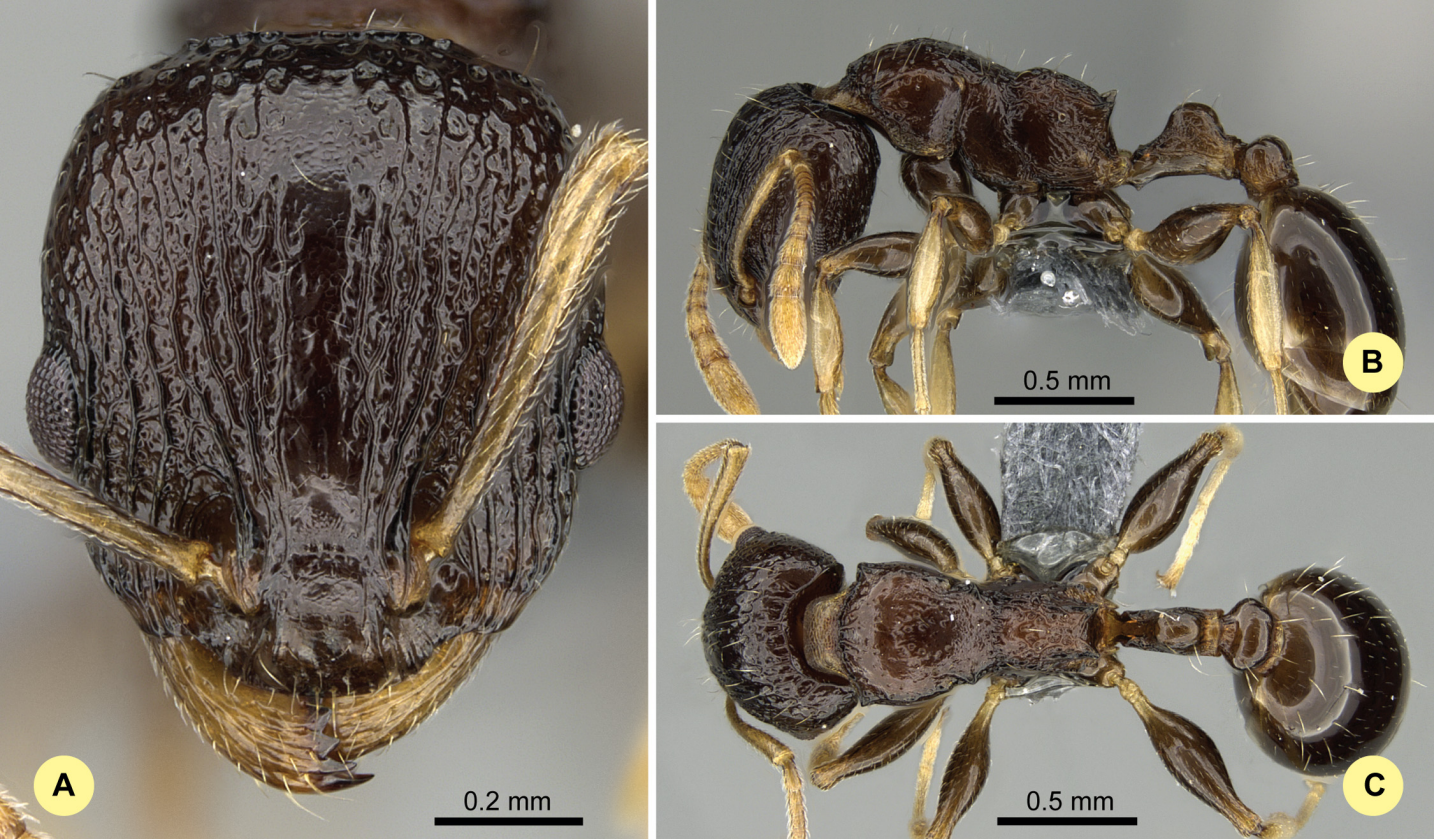
*Nesomyrmex modestus* sp. n. holotype worker (CASENT0393175). (A) Head of the holotype worker in full-face view. (B) Lateral view of the body. (C) Dorsal view of the body.

urn:lsid:zoobank.org:act:A8B6069A-2FD3-4CEA-AC0D-DB36127037B0

(Figs 3 and 10A–10C, Table 1.)

#### Type material investigated

Holotype worker: MADGAGASCAR: Prov. Toliara, Anosy Region, Anosyenne Mts, 29.33 km NW Manantenina, Madagascar, 540 m, -24.13993 N, 47.07418 E, 21.ii.2015, collection code: BLF36218; CASENT0393175, B.L.Fisher, F.A.Esteves et al. (CAS, CASENT0393175);

Paratypes: 13 workers with the same label data with the holotype under CASENT codes: CASENT0393167, BLF36224, (1w, CAS); CASENT0393168, BLF36224, (1w, CAS); CASENT0393169, BLF36224, (1w, CAS); CASENT0393170, BLF36224, (1w, CAS); CASENT0393171, BLF36224, (1w, CAS); CASENT0393172, BLF36224, (1w, CAS); CASENT0393173, BLF36224, (1w, 1Q, CAS); CASENT0393176, BLF36218, (1w, CAS); CASENT0393177, BLF36218, (1w, CAS); CASENT0393178, BLF36218, (1w, CAS); CASENT0393179, BLF36218, (1w, CAS); CASENT0393180, BLF36218, (1w, CAS); CASENT0393181, BLF36218, (1w, 1Q, CAS);

The list of 18 worker individuals belonging to 18 nest samples morphometrically investigated is given in S1 Table.

#### Diagnosis

See in key.

#### Description of workers

Body color: black. Body color pattern: concolorous. Absolute cephalic size: 796 μm [743, 834]. Cephalic length vs. maximum width of head capsule (CL/CWb): 1.216 [1.179, 1.256]. Postocular distance vs. cephalic length (PoOc/CL): 0.453 [0.438, 0.466]. Postocular sides of cranium contour, anterior view orientation: converging posteriorly. Postocular sides of cranium contour, anterior view shape: convex. Vertex contour line in anterior view shape: straight; feebly convex. Vertex sculpture: main sculpture areolate, ground sculpture smooth. Gena contour line in anterior view shape: convex. Gena contour from anterior view orientation: strongly converging. Gena sculpture: rugoso-reticulate with feeble areolate ground sculpture; rugoso-reticulate with areolate ground sculpture. Concentric carinae laterally surrounding antennal foramen: present. Eye length vs. absolute cephalic size (EL/CS): 0.234 [0.217, 0.25]. Frontal carina distance vs. absolute cephalic size (FRS/CS): 0.295 [0.287, 0.307]. Longitudinal carinae on median region of frons count: present. Smooth median region on frons count: present. Antennomere count: 12. Scape length vs. absolute cephalic size (SL/CS): 0.771 [0.74, 0.808]. Median clypeal notch: present. Median carina of clypeus count: absent. Spine length vs. absolute cephalic size (SPST/CS): 0.236 [0.22, 0.254]. Minimum spine distance vs. absolute cephalic size (SPBA/CS): 0.236 [0.225, 0.248]. Apical spine distance vs. absolute cephalic size (SPTI/CS): 0.251 [0.226, 0.269]. Propodeal spine shape: triangular, blunt. Apical distance of pronotal spines vs. absolute cephalic size (PSTI/CS): 0.657 [0.636, 0.686]. Metanotal depression count: present. Dorsal region of mesosoma sculpture: smooth ground sculpture superimposed by feeble areolate main sculpture. Lateral region of pronotum sculpture: inconspicuous areolate ground sculpture, main sculpture dispersed costate. Mesopleuron sculpture: smooth ground sculpture, superimposed by dispersed rugulae. Metapleuron sculpture: fine areolate ground sculpture, superimposed by dispersed rugulae. Petiole width vs. absolute cephalic size (PEW/CS): 0.223 [0.213, 0.238]. Dorsal region of petiole sculpture: ground sculpture smooth, main sculpture absent. Postpetiole width vs. absolute cephalic size (PPW/CS): 0.341 [0.326, 0.358]. Dorsal region of postpetiole sculpture: ground sculpture smooth, main sculpture absent.

#### Etymology

The name (modestus = moderate) refers to the moderately coarse surface sculpturing of this species.

#### Distribution

This species is known to occur in rainforests between elevation of 520 m and 1325 m (mean: 514 m) in the southwestern part of Madagascar (Fig 3).

### Nesomyrmex reticulatus Csősz & Fisher sp. n.

**Fig 11:**
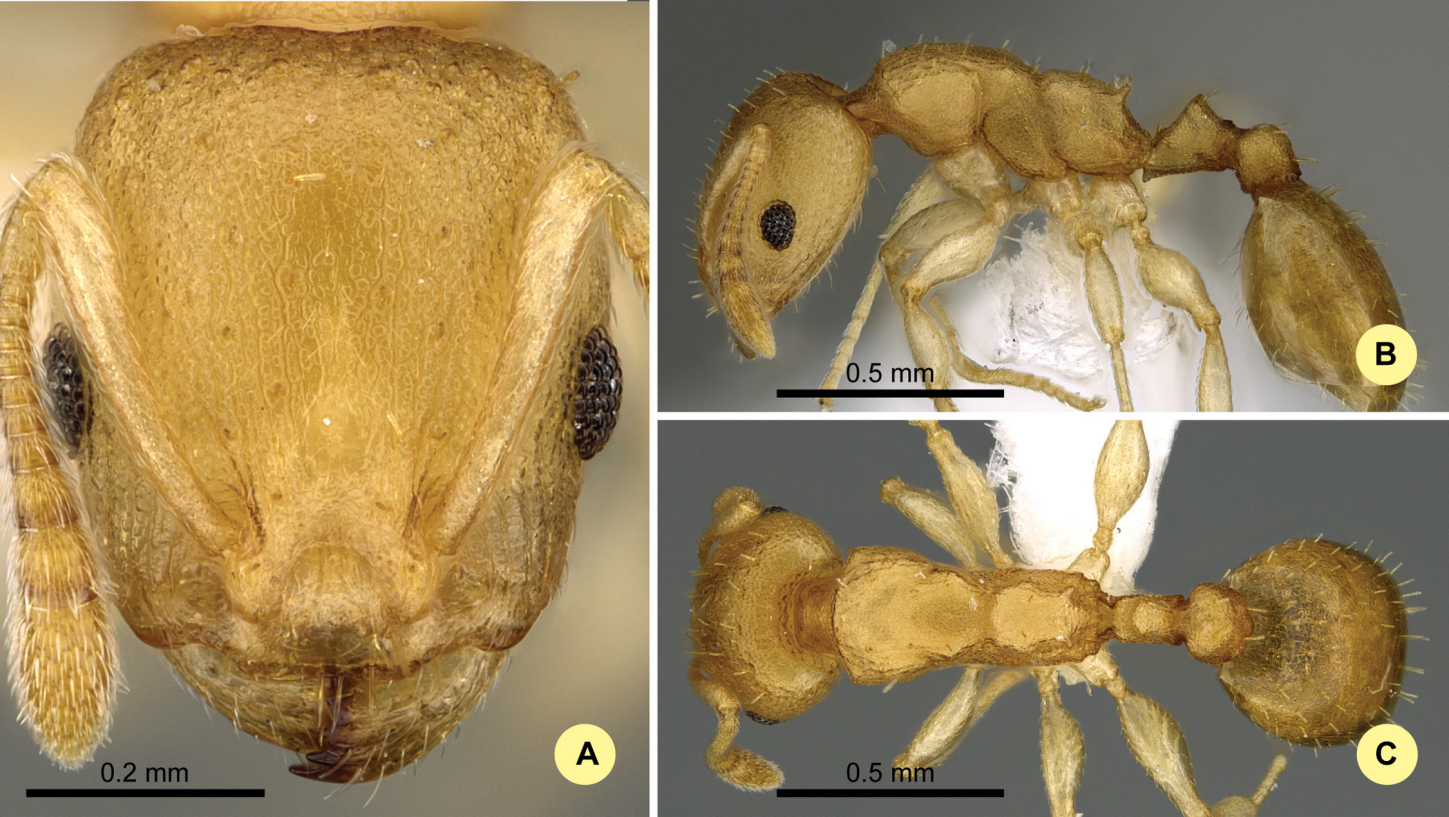
*Nesomyrmex reticulatus* sp. n. holotype worker (CASENT0418497). (A) Head of the holotype worker in full-face view. (B) Lateral view of the body. (C) Dorsal view of the body.

urn:lsid:zoobank.org:act:3E5E26A6-6795-45AA-A051-2AFDAF57E6E5

(Figs 3 and 11A–11C, Table 1.)

#### Type material investigated

Holotype worker: MADGAGASCAR: Prov. Toliara, Forêt de Kirindy, 15.5 km 64° ENE Marofandilia, 100 m, -20.045 N, 44.66222 E, 28.xi.2001, collection code: BLF04604; CASENT0418497, Fisher-Griswold Arthropod Team, beating low vegetation tropical dry forest (CAS, CASENT0418497);

Paratypes: 3 workers and a gyne with the same label data with the holotype under CASENT codes: CASENT0473935, BLF04605, (1w, CAS); CASENT0418443, BLF04604, (1Q, CAS); CASENT0418447, BLF04604, (1w, CAS); CASENT0418482, BLF04604, (1w, CAS);

The list of 7 worker individuals belonging to 7 nest samples morphometrically investigated is given in S1 Table.

#### Diagnosis

See in key.

#### Description of workers

Body color: yellow; brown. Body color pattern: concolorous. Absolute cephalic size: 511 μm [472, 545]. Cephalic length vs. maximum width of head capsule (CL/CWb): 1.151 [1.125, 1.18]. Postocular distance vs. cephalic length (PoOc/CL): 0.462 [0.449, 0.476]. Postocular sides of cranium contour, anterior view orientation: converging posteriorly. Postocular sides of cranium contour, anterior view shape: strongly convex. Vertex contour line in anterior view shape: slightly concave. Vertex sculpture: main sculpture homogeneously forked costate, ground sculpture areolate. Gena contour line in anterior view shape: convex. Gena contour from anterior view orientation: strongly converging. Gena sculpture: rugoso-reticulate with areolate ground sculpture. Concentric carinae laterally surrounding antennal foramen: present. Eye length vs. absolute cephalic size (EL/CS): 0.238 [0.224, 0.254]. Frontal carina distance vs. absolute cephalic size (FRS/CS): 0.322 [0.310, 0.335]. Longitudinal carinae on median region of frons: absent. Smooth median region on frons count: absent. Antennomere count: 12. Scape length vs. absolute cephalic size (SL/CS): 0.704 [0.679, 0.72]. Median clypeal notch: present. Median carina of clypeus: absent. Spine length vs. absolute cephalic size (SPST/CS): 0.236 [0.224, 0.257]. Minimum spine distance vs. absolute cephalic size (SPBA/CS): 0.254 [0.235, 0.275]. Apical spine distance vs. absolute cephalic size (SPTI/CS): 0.267 [0.244, 0.284]. Propodeal spine shape: straight; triangular, blunt. Apical distance of pronotal spines vs. absolute cephalic size (PSTI/CS): 0.629 [0.608, 0.65]. Metanotal depression: present. Dorsal region of mesosoma sculpture: areolate ground sculpture, superimposed by dispersed rugae. Lateral region of pronotum sculpture: areolate ground sculpture, main sculpture dispersed costate. Mesopleuron sculpture: areolate ground sculpture superimposed by dispersed rugulae. Metapleuron sculpture: areolate ground sculpture superimposed by dispersed rugulae. Petiole width vs. absolute cephalic size (PEW/CS): 0.235 [0.222, 0.242]. Dorsal region of petiole sculpture: ground sculpture areolate, main sculpture absent. Postpetiole width vs. absolute cephalic size (PPW/CS): 0.37 [0.339, 0.386]. Dorsal region of postpetiole sculpture: ground sculpture areolate, main sculpture absent.

#### Etymology

The name refers to the fine, micro-reticulate body sculpturing.

#### Distribution

This species is known to occur in dry forests in lowlands between 50 m and 130 m (mean: 94 m) of the western and southern coasts of Madagascar (Fig 3).

### Nesomyrmex retusispinosus (Forel, 1892)

**Fig 12:**
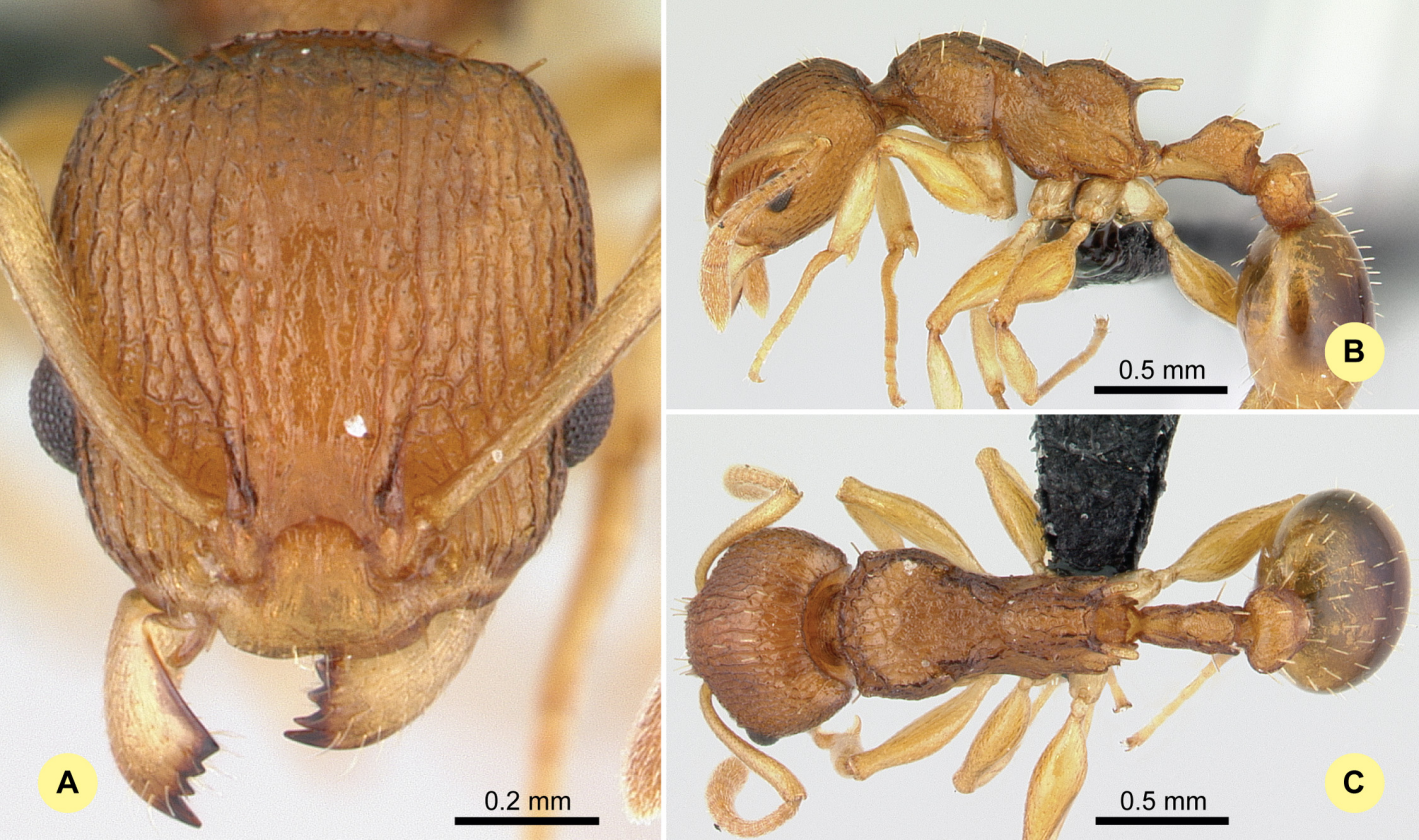
*Nesomyrmex retusispinosus* (Forel, 1892) non-type worker (CASENT0060902). (A) Head of the worker in full-face view. (B) Lateral view of the body. (C) Dorsal view of the body.

(Figs 3 and 12A–12C, Table 1.)

#### Type material investigated

Holotype worker: “L. retusispinosus, type, Forel, For d' andrangoloaca [Antananarivo, -18.91 N, 47.55 E], Madagascar (Sikora)”, (MHNG, CASENT0101616);

The list of 9 worker individuals belonging to 9 nest samples morphometrically investigated is given in S1 Table.

#### Diagnosis

See in key.

#### Description of workers

Body color: yellow; brown. Body color pattern: concolorous. Absolute cephalic size: 812 μm [731, 921]. Cephalic length vs. maximum width of head capsule (CL/CWb): 1.17 [1.07, 1.233]. Postocular distance vs. cephalic length (PoOc/CL): 0.483 [0.464, 0.498]. Postocular sides of cranium contour, anterior view orientation: converging posteriorly. Postocular sides of cranium contour, anterior view shape: convex. Vertex contour line in anterior view shape: straight; feebly convex. Vertex sculpture: main sculpture rugoso-reticulate, ground sculpture areolate. Gena contour line in anterior view shape: convex. Gena contour from anterior view orientation: strongly converging. Gena sculpture: rugoso-reticulate with areolate ground sculpture. Concentric carinae laterally surrounding antennal foramen: present. Eye length vs. absolute cephalic size (EL/CS): 0.237 [0.225, 0.251]. Frontal carina distance vs. absolute cephalic size (FRS/CS): 0.294 [0.285, 0.302]. Longitudinal carinae on median region of frons: present. Smooth median region on frons: absent. Antennomere count: 12. Scape length vs. absolute cephalic size (SL/CS): 0.838 [0.812, 0.862]. Median clypeal notch: present. Median carina of clypeus: present or absent. Spine length vs. absolute cephalic size (SPST/CS): 0.43 [0.4, 0.459]. Minimum spine distance vs. absolute cephalic size (SPBA/CS): 0.24 [0.219, 0.259]. Apical spine distance vs. absolute cephalic size (SPTI/CS): 0.322 [0.293, 0.332]. Propodeal spine shape: slightly or strongly bent. Apical distance of pronotal spines vs. absolute cephalic size (PSTI/CS): 0.659 [0.621, 0.678]. Metanotal depression: present. Dorsal region of mesosoma sculpture: rugulose with areolate ground sculpture. Lateral region of pronotum sculpture: areolate ground sculpture, main sculpture rugoso-reticulate. Mesopleuron sculpture: areolate ground sculpture, superimposed by dispersed rugae. Metapleuron sculpture: areolate ground sculpture, superimposed by dispersed rugae. Petiole width vs. absolute cephalic size (PEW/CS): 0.24 [0.219, 0.262]. Dorsal region of petiole sculpture: ground sculpture areolate, main sculpture dispersed rugose. Postpetiole width vs. absolute cephalic size (PPW/CS): 0.39 [0.358, 0.415]. Dorsal region of postpetiole sculpture: ground sculpture areolate, main sculpture dispersed rugose.

#### Distribution

This species is known to occur in rain forests and montane forests in lowlands between 918 m and 1080 m in central Madagascar (Fig 3).

### *Nesomyrmex rugosus* Csősz & Fisher sp. n.

**Fig 13:**
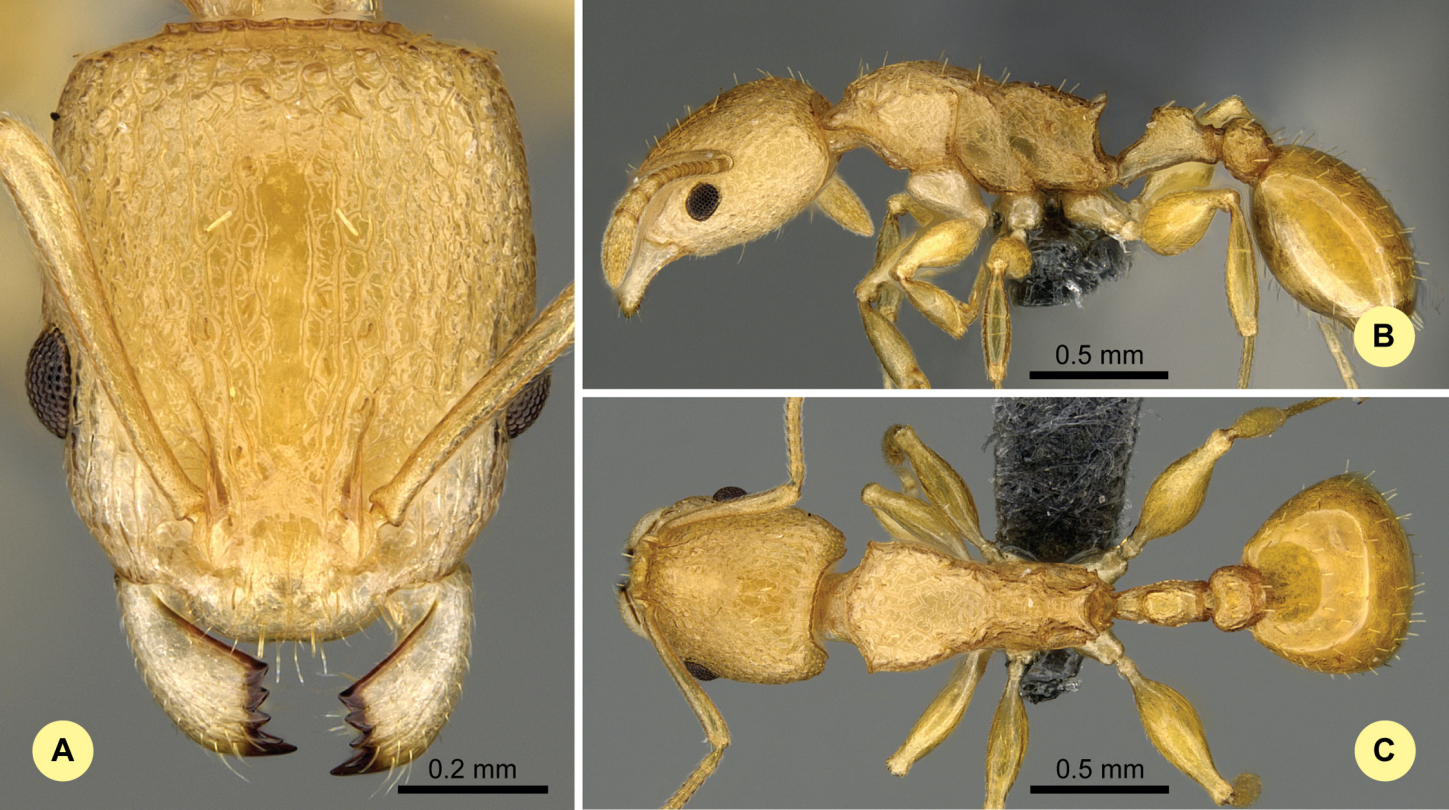
*Nesomyrmex rugosus* sp. n. holotype worker (CASENT0374465). (A) Head of the holotype worker in full-face view. (B) Lateral view of the body. (C) Dorsal view of the body.

urn:lsid:zoobank.org:act:C39EB560-EC60-4720-B6D8-95CE88C75A4D

(Figs 3 and 13A–13C, Table 1.)

#### Type material investigated

Holotype worker: MADGAGASCAR: Prov. Antsisarana, Masoala National Park, 250 m, -15.33058 N, 50.30279 E, 13.iii.2014, collection code: BLF33157; CASENT0374465, Fisher et al. (CAS, CASENT0374465);

Paratypes: 2 workers, a gyne and a male with the same label data with the holotype under CASENT codes: CASENT763560, BLF33157, (1q, CAS), CASENT0374466, BLF33157, (1w, 1m, CAS); CASENT0377059, BLF32874, (1w, CAS);

The list of 24 worker individuals belonging to 23 nest samples morphometrically investigated is given in S1 Table.

#### Diagnosis

See in key.

#### Description of workers

Body color: yellow to brown. Body color pattern: concolorous. Absolute cephalic size: 747 μm [650, 839]. Cephalic length vs. maximum width of head capsule (CL/CWb): 1.239 [1.189, 1.277]. Postocular distance vs. cephalic length (PoOc/CL): 0.467 [0.448, 0.493]. Postocular sides of cranium contour, anterior view orientation: converging posteriorly. Postocular sides of cranium contour, anterior view shape: convex. Vertex contour line in anterior view shape: straight or feebly convex. Vertex sculpture: main sculpture rugoso-reticulate, ground sculpture smooth. Gena contour line in anterior view shape: convex. Genae contour from anterior view orientation: strongly converging. Gena sculpture: rugoso-reticulate with feeble areolate ground sculpture. Concentric carinae laterally surrounding antennal foramen: present. Eye length vs. absolute cephalic size (EL/CS): 0.226 [0.21, 0.235]. Frontal carina distance vs. absolute cephalic size (FRS/CS): 0.292 [0.277, 0.312]. Longitudinal carinae on median region of frons: present. Smooth median region on frons: absent. Antennomere count: 12. Scape length vs. absolute cephalic size (SL/CS): 0.816 [0.784, 0.856]. Median clypeal notch count: present. Median carina of clypeus: absent. Spine length vs. absolute cephalic size (SPST/CS): 0.288 [0.253, 0.352]. Minimum spine distance vs. absolute cephalic size (SPBA/CS): 0.221 [0.182, 0.256]. Apical spine distance vs. absolute cephalic size (SPTI/CS): 0.257 [0.191, 0.31]. Propodeal spine shape: straight. Apical distance of pronotal spines vs. absolute cephalic size (PSTI/CS): 0.635 [0.6, 0.692]. Metanotal depression: present. Dorsal region of mesosoma sculpture: rugose with smooth ground sculpture. Lateral region of pronotum sculpture: inconspicuously areolate ground sculpture, main sculpture rugoso-reticulate. Mesopleuron sculpture: smooth ground sculpture, superimposed by dispersed rugulae. Metapleuron sculpture: areolate ground sculpture, superimposed by dispersed rugae. Petiole width vs. absolute cephalic size (PEW/CS): 0.231 [0.2, 0.278]. Dorsal region of petiole sculpture: ground sculpture inconspicuously areolate, main sculpture rogoso-reticulate. Postpetiole width vs. absolute cephalic size (PPW/CS): 0.345 [0.308, 0.376]. Dorsal region of postpetiole sculpture: ground sculpture smooth, main sculpture dispersed rugose.

#### Etymology

The name refers to the coarse, rugose body sculpturing.

#### Distribution

This species is known to occur in rainforests between elevation of 200 m and 520 m (mean: 390 m) of the eastern and southern coasts of Madagascar (Fig 3).

### Nesomyrmex sikorai (Emery, 1896)

**Fig 14:**
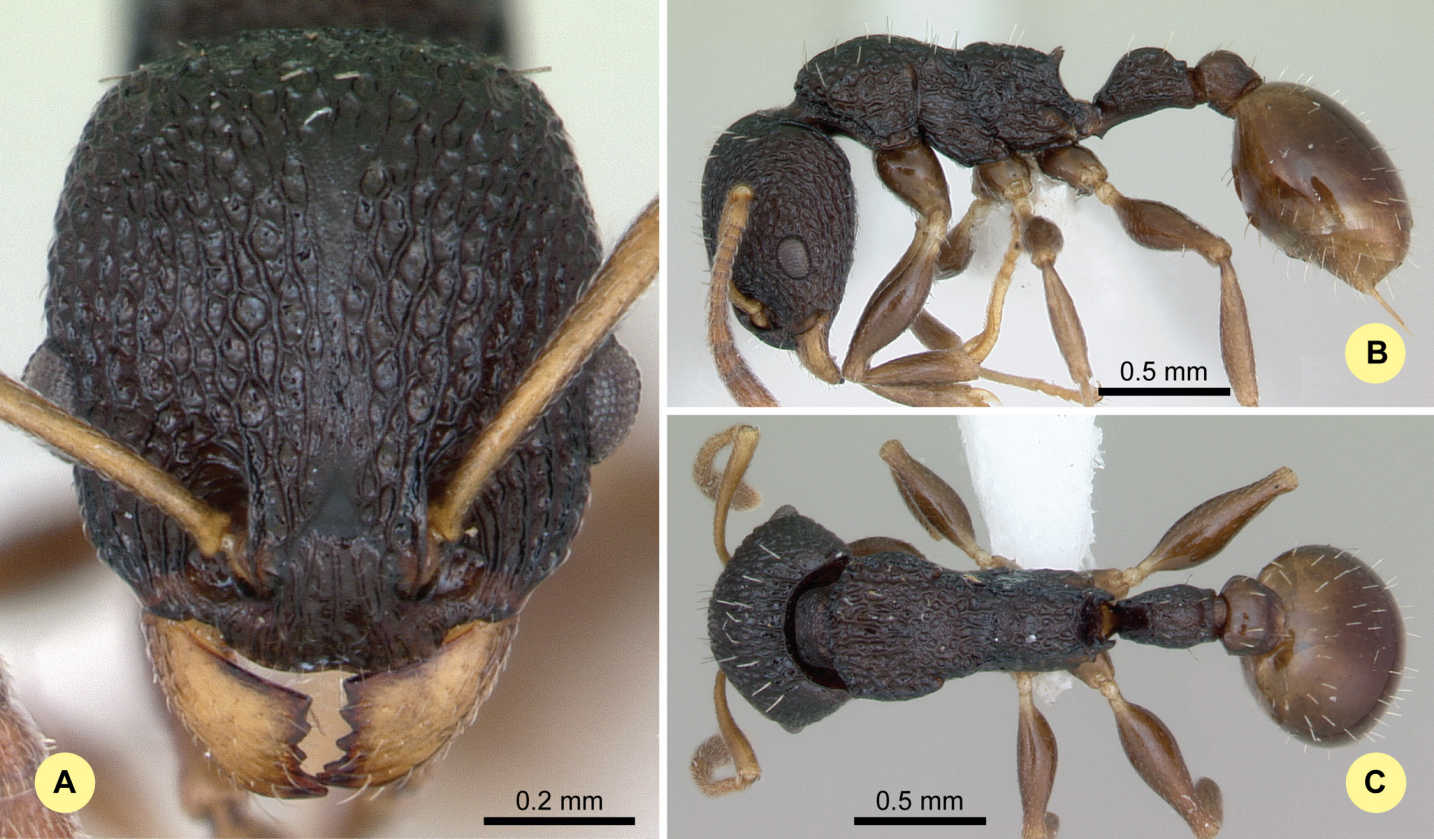
*Nesomyrmex sikorai* (Emery, 1896) non-type worker (CASENT0003120). (A) Head of the worker in full-face view. (B) Lateral view of the body (C) Dorsal view of the body.

(Figs 3 and 14A–14C, Table 1.)

#### Type material investigated

Holotype worker: “Leptothorax sikorai n. sp., Imerina [Antananarivo, -18.91 N, 47.55 E], Sikora” TYPUS (MHNG, CASENT0102064). Note: the type was not available for morphometric investigation; high-quality AntWeb images were used for comparison.

The list of 36 worker individuals belonging to 28 nest samples morphometrically investigated is given in S1 Table.

#### Diagnosis

See in key.

#### Description of workers

Body color: black. Body color pattern: concolorous. Absolute cephalic size: 822 μm [748, 890]. Cephalic length vs. maximum width of head capsule (CL/CWb): 1.169 [1.126, 1.226]. Postocular distance vs. cephalic length (PoOc/CL): 0.488 [0.451, 0.511]. Postocular sides of cranium contour, anterior view orientation: converging posteriorly. Postocular sides of cranium contour, anterior view shape: strongly convex. Vertex contour line in anterior view shape: straight to feebly convex. Vertex sculpture: main sculpture areolate, ground sculpture areolate. Gena contour line in anterior view shape: convex. Gena contour from anterior view orientation: strongly converging. Gena sculpture: rugoso-reticulate with areolate ground sculpture. Concentric carinae laterally surrounding antennal foramen: present. Eye length vs. absolute cephalic size (EL/CS): 0.212 [0.201, 0.224]. Frontal carina distance vs. absolute cephalic size (FRS/CS): 0.292 [0.276, 0.312]. Longitudinal carinae on median region of frons: present. Smooth median region on frons: absent. Antennomere count: 12. Scape length vs. absolute cephalic size (SL/CS): 0.828 [0.78, 0.862]. Median clypeal notch: present. Median carina of clypeus: present or absent. Spine length vs. absolute cephalic size (SPST/CS): 0.269 [0.221, 0.301]. Minimum spine distance vs. absolute cephalic size (SPBA/CS): 0.253 [0.215, 0.287]. Apical spine distance vs. absolute cephalic size (SPTI/CS): 0.299 [0.256, 0.341]. Propodeal spine shape: straight; triangular, blunt. Apical distance of pronotal spines vs. absolute cephalic size (PSTI/CS): 0.665 [0.636, 0.691]. Metanotal depression: present. Dorsal region of mesosoma sculpture: areolate main sculpture, interstices areolate. Lateral region of pronotum sculpture: areolate ground sculpture, main sculpture areolate. Mesopleuron sculpture: areolate ground sculpture, superimposed by dispersed rugae. Metapleuron sculpture: areolate ground sculpture, superimposed by coarse rugae. Petiole width vs. absolute cephalic size (PEW/CS): 0.269 [0.228, 0.326]. Dorsal region of petiole sculpture: ground sculpture areolate, main sculpture rugoso-reticulate. Postpetiole width vs. absolute cephalic size (PPW/CS): 0.37 [0.348, 0.391]. Dorsal region of postpetiole sculpture: ground sculpture smooth, main sculpture absent.

#### Distribution

This species is known to occur in montane rainforests between elevations of 200 m and 520 m in central Madagascar (Fig 3).

### *Nesomyrmex striatus* Csősz & Fisher sp. n.

**Fig 15:**
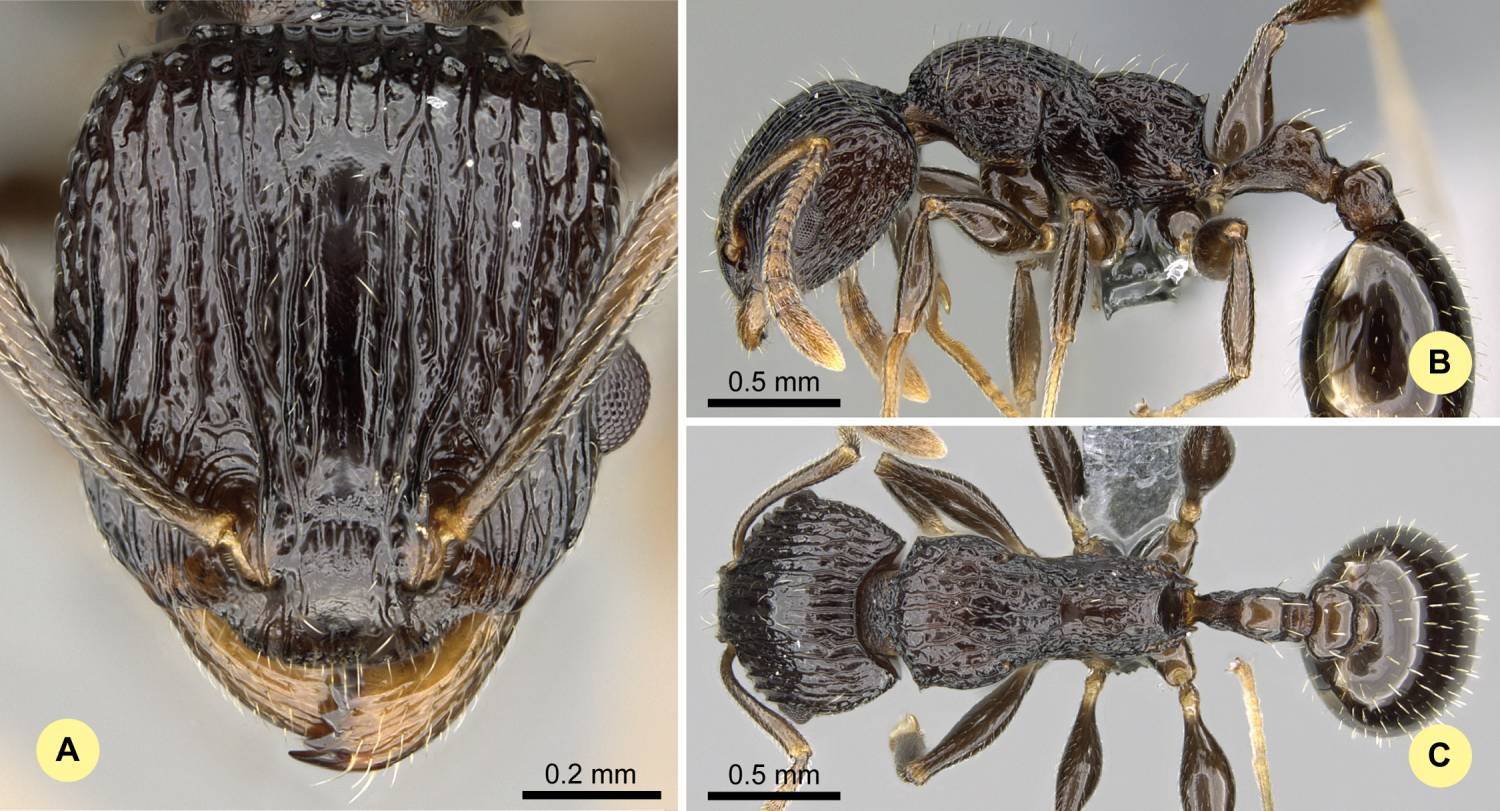
*Nesomyrmex striatus* sp. n. holotype worker (CASENT0393121). (A) Head of the holotype worker in full-face view. (B) Lateral view of the body. (C) Dorsal view of the body.

urn:lsid:zoobank.org:act:538BA1CB-262A-492E-86E5-035513668BE9

(Figs 3 and 15A–15C, Table 1.)

#### Type material investigated

Holotype worker: MADGAGASCAR: Prov. Toliara, Anosy Region, Anosyenne Mts., 31.2 km NW Manantenina, 1125 m, -24.13894 N, 47.06804 E, 26.ii.2015, collection code: BLF36533; CASENT0393121, Fisher et al. (CAS, CASENT0393121);

Paratypes: 5 workers and a male with the same label data with the holotype under CASENT codes: CASENT0393116, BLF36533, (1w, 1m, CAS); CASENT0393117, BLF36533, (1w, CAS); CASENT0393118, BLF36533, (1w, CAS); CASENT0393119, BLF36533, (1w, CAS); CASENT0393120, BLF36533, (1w, CAS);

The list of 9 worker individuals belonging to 9 nest samples morphometrically investigated is given in S1 Table.

#### Diagnosis

See in key.

#### Description of workers

Body color: black. Body color pattern: concolorous. Absolute cephalic size: 842 μm [775, 889]. Cephalic length vs. Maximum width of head capsule (CL/CWb): 1.155 [1.117, 1.192]. Postocular distance vs. cephalic length (PoOc/CL): 0.460 [0.441, 0.472]. Postocular sides of cranium contour, anterior view orientation: converging posteriorly. Postocular sides of cranium contour, anterior view shape: convex. Vertex contour line in anterior view shape: straight to feebly convex. Vertex sculpture: main sculpture parallel costate, ground sculpture smooth. Gena contour line in anterior view shape: convex. Gena contour from anterior view orientation: strongly converging. Gena sculpture: rugoso-reticulate with feeble areolate ground sculpture. Concentric carinae laterally surrounding antennal foramen: present. Eye length vs. absolute cephalic size (EL/CS): 0.219 [0.209, 0.232]. Frontal carina distance vs. absolute cephalic size (FRS/CS): 0.288 [0.282, 0.294]. Longitudinal carinae on median region of frons: present. Smooth median region on frons: present. Antennomere count: 12. Scape length vs. absolute cephalic size (SL/CS): 0.794 [0.779, 0.813]. Median clypeal notch count: present. Median carina of clypeus: absent. Spine length vs. absolute cephalic size (SPST/CS): 0.241 [0.219, 0.26]. Minimum spine distance vs. absolute cephalic size (SPBA/CS): 0.242 [0.204, 0.26]. Apical spine distance vs. absolute cephalic size (SPTI/CS): 0.244 [0.191, 0.266]. Propodeal spine shape: curving upward. Apical distance of pronotal spines vs. absolute cephalic size (PSTI/CS): 0.666 [0.649, 0.686]. Metanotal depression: present. Dorsal region of mesosoma sculpture: costate with smooth ground sculpture. Lateral region of pronotum sculpture: areolate ground sculpture, main sculpture areolate. Mesopleuron sculpture: smooth ground sculpture, superimposed by dispersed rugulae. Metapleuron sculpture: fine areolate ground sculpture, superimposed by coarse rugae. Petiole width vs. absolute cephalic size (PEW/CS): 0.243 [0.229, 0.253]. Dorsal region of petiole sculpture: ground sculpture smooth, main sculpture absent. Postpetiole width vs. absolute cephalic size (PPW/CS): 0.362 [0.351, 0.373]. Dorsal region of postpetiole sculpture: ground sculpture smooth, main sculpture absent.

#### Etymology

The name refers to the coarse, striate/costate surface sculpturing on the head and mesosoma.

#### Distribution

This species is known to occur in montane rainforests between elevations of 900 m and 1125 m (mean: 1054 m) of the southeastern part of Madagascar (Fig 3).

### Nesomyrmex tamatavensis Csősz & Fisher sp. n.

**Fig 16:**
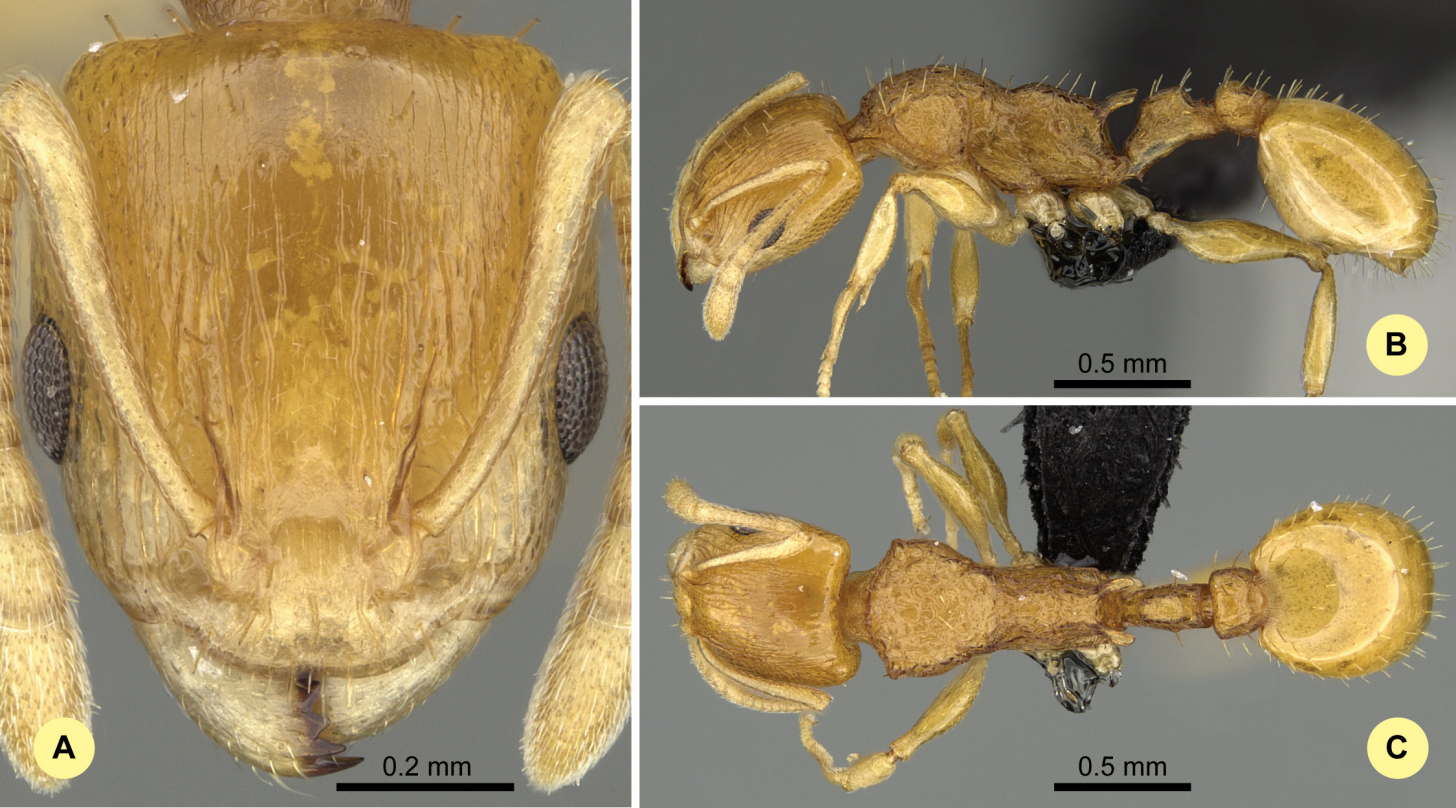
*Nesomyrmex tamatavensis* sp. n. holotype worker (CASENT0496292). (A) Head of the holotype worker in full-face view. (B) Lateral view of the body. (C) Dorsal view of the body.

urn:lsid:zoobank.org:act:A67F58B6-502A-4798-B638-6FB35C78BD27

(Figs 3 and 16A–16C, Table 1.)

#### Type material investigated

Holotype worker: MADGAGASCAR: Prov. Toamasina, Montagne d'Akirindro 7.6 km 341° NNW Ambinanitelo, 600 m, -15.28833 N, 49.54833 E, 17.iii.2003, collection code: BLF8390; CASENT0496292, Fisher et al., ex dead twig above ground, rainforest (CAS, CASENT0496292);

Paratypes: 5 workers and a male with the same label data with the holotype with CASENT codes: CASENT0763559, BLF8390, (2w, CAS); CASENT0496293, BLF8390, (3w, CAS); CASENT0496294, BLF8390, (3w, CAS); CASENT0496295, BLF8390, (1q, 1m, CAS);

The list of 41 worker individuals belonging to 32 nest samples morphometrically investigated is given in S1 Table.

#### Diagnosis

See in key.

#### Description of workers

Body color: yellow to brown. Body color pattern: concolorous. Absolute cephalic size: 699 μm [634, 750]. Cephalic length vs. maximum width of head capsule (CL/CWb): 1.155 [1.119, 1.182]. Postocular distance vs. cephalic length (PoOc/CL): 0.433 [0.411, 0.449]. Postocular sides of cranium contour, anterior view orientation: converging posteriorly. Postocular sides of cranium contour, anterior view shape: convex. Vertex contour line in anterior view shape: straight to feebly convex. Vertex sculpture: main sculpture inconspicuous, ground sculpture smooth. Gena contour line in anterior view shape: convex. Gena contour from anterior view orientation: strongly converging. Gena sculpture: rugoso-reticulate with feeble areolate ground sculpture. Concentric carinae laterally surrounding antennal foramen: present. Eye length vs. absolute cephalic size (EL/CS): 0.258 [0.243, 0.284]. Frontal carina distance vs. absolute cephalic size (FRS/CS): 0.329 [0.318, 0.347]. Longitudinal carinae on median region of frons: present or absent. Smooth median region on frons: present. Antennomere count: 12. Scape length vs. absolute cephalic size (SL/CS): 0.798 [0.773, 0.832]. Median clypeal notch: present. Median carina of clypeus: absent. Spine length vs. absolute cephalic size (SPST/CS): 0.388 [0.364, 0.43]. Minimum spine distance vs. absolute cephalic size (SPBA/CS): 0.244 [0.221, 0.282]. Apical spine distance vs. absolute cephalic size (SPTI/CS): 0.303 [0.273, 0.35]. Propodeal spine shape: strongly bent. Apical distance of pronotal spines vs. absolute cephalic size (PSTI/CS): 0.739 [0.708, 0.78]. Metanotal depression: present. Dorsal region of mesosoma sculpture: rugose with smooth ground sculpture. Lateral region of pronotum sculpture: inconspicuously areolate ground sculpture, main sculpture rugoso-reticulate. Mesopleuron sculpture: smooth ground sculpture, superimposed by dispersed rugulae. Metapleuron sculpture: fine areolate ground sculpture, superimposed by dispersed rugulae. Petiole width vs. absolute cephalic size (PEW/CS): 0.243 [0.208, 0.27]. Dorsal region of petiole sculpture: ground sculpture inconspicuously areolate, main sculpture rugoso-reticulate. Postpetiole width vs. absolute cephalic size (PPW/CS): 0.363 [0.338, 0.385]. Dorsal region of postpetiole sculpture: ground sculpture smooth, main sculpture dispersed rugose.

#### Etymology

Tamatave is the French name of Toamasina, the capital of the Atsinanana region, where this species is abundant.

#### Distribution

This species is known to occur in rainforests from sea level to 1100 m predominantly in the northeastern part of Madagascar (Fig 3). The only known locality of this species in the southwestern Toliara region (Forêt Classée d'Analavelona, NNW Mahaboboka) is in an isolated rainforest surrounded by dry forest; this rainforest is a relic of the formerly widespread rainforest now found primarily in the east.

## Supporting Information

S1 Appendix Extracted character matrix for each taxon treated in this revisionary work generated by the MX database. (NEX)

S2 Appendix R script of NC clustering and method PART implementing cluster methods “hclust” and “kmeans”. Mark dendrogram function mapping the results of partitioning algorithm PART on the dendrogram is also added.
(TXT)

S1 Table List of morphometrically investigated samples. Unique CASENT number for pinned samples, locality, geographic coordinates (E, N) in decimal format, altitude (ALT) in meters a.s.l., collector’s name, date and number of specimens investigated bearing the given CASENT number are provided. HT = Holotype, PT = paratype(s). All samples collected in Toliara administrative region, Madagascar, and deposited at the California Academy of Sciences (CAS).
(XLSX)

S2 Table URI table for morphometric characters and Hymenoptera-specific terminology of morphological statements used in descriptions, identification key, and diagnoses are mapped to classes in phenotype-relevant ontologies. (DOCX)

S3 Table Morphometric data of 22 continuous morphometric traits of 227 individuals is given in μm. CASENT code (casent), final species hypothesis (species), geographic coordinates (long, lat) and the name format as samples appear on the dendrogram (dendro-name) are also provided in the table. HT = Holotype, PT = paratype(s).
(CSV)

## References

[1] http://www.commondreams.org/news/2013/05/28/un-accelerating-biodiversity-loss-fundamental-threat-survival-humankind.

[2] Ceballos G, Ehrlich PR, Barnosky AD, García A, Pringle RM, Palmer TM. Accelerated modern human–induced species losses: Entering the sixth mass extinction. Science Advances. 2015; 1: e1400253 10.1126/sciadv.1400253 26601195 PMC4640606

[3] Klingenberg CP, Monteiro LR. Distances and directions in multidimensional shape spaces: implications for morphometric applications. Syst Biol. 2005; 54: 678–688. 16126663 10.1080/10635150590947258

[4] Ezard TH Pearson PN, Purvis A. Algorithmic approaches to aid species’ delimitation in multidimensional morphospace. BMC Evol Biol. 2010; 10: 1–11. 20540735 10.1186/1471-2148-10-175 PMC2898690

[5] Baur H, Leuenberger C. Analysis of ratios in multivariate morphometry. Syst Biol. 2011; 60: 813–825. 10.1093/sysbio/syr061 21828084 PMC3193766

[6] Derkarabetian S, Hedin M. Integrative taxonomy and species delimitation in harvestmen: A revision of the western North American genus *Sclerobunus* (Opiliones: Laniatores: Travunioidea) PLoS ONE 2014; 9: e104982 10.1371/journal.pone.0104982 25144370 PMC4140732

[7] Seifert B, Ritz M, Csősz S. Application of exploratory data analyses opens a new perspective in morphology-based alpha-taxonomy of eusocial organisms. Myrmecol News. 2014; 19: 1–15.

[8] Baur H, Kranz-Baltensperger Y, Cruaud A, Rasplus JY, Timokhov AV, Gokhman VE. Morphometric analysis and taxonomic revision of *Anisopteromalus* Ruschka (Hymenoptera: Chalcidoidea: Pteromalidae)—an integrative approach. Syst Entomol. 2014; 39: 691–709. 26074661 10.1111/syen.12081 PMC4459240

[9] Seifert B, Csősz S. *Temnothorax crasecundus* sp. n.—a cryptic Eurocaucasian ant species (Hymenoptera, Formicidae) discovered by Nest Centroid Clustering. ZooKeys 2015; 479: 37–64. 10.3897/zookeys.479.8510 25685016 PMC4319063

[10] Nilsen G, Borgan Ø, LiestØl K, Lingjærde OC. Identifying clusters in genomics data by recursive partitioning. Stat Appl Genet Mol Biol. 2013; 12: 637–652. 10.1515/sagmb-2013-0016 23942354

[11] Csősz S, Fisher BL. Diagnostic survey of Malagasy *Nesomyrmex* species-groups and revision of *hafahafa* group species via morphology based cluster delimitation protocol. ZooKeys 2015; 526: 19–59. 10.3897/zookeys.526.6037 26487823 PMC4607843

[12] Csősz S, Seifert B, Müller B, Trindl A, Schulz A, Heinze J. Cryptic diversity in the Mediterranean *Temnothorax lichtensteini* species-complex (Hymenoptera: Formicidae). Org Div Evol. 2014; 14: 75–88.

[13] Guillem RM, Drijfhout F, Martin SJ. Chemical deception among ant social parasites. Current Zoology 2014; 60: 62–75.

[14] Wachter GA, Muster C, Arthofer W, Raspotnig G, Föttinger P, Komposch C et al Taking the discovery approach in integrative taxonomy: decrypting a complex of narrow-endemic Alpine harvestmen (Opiliones: Phalangiidae: *Megabunus*). Mol Ecol. 2015; 24: 863–889. 10.1111/mec.13077 25583278

[15] Tibshirani R, Walther G, Hastie T () Estimating the number of clusters in a data set via the gap statistic. J R Stat Soc. 2001; 63: 411–423.

[16] Evenhuis NL. The insect and spider collections of the world website. Available: http://hbs.bishopmuseum.org/codens/. Accessed 3 Mar 2014.

[17] R Core Team. R: A language and environment for statistical computing. R Foundation for Statistical Computing, Vienna, Austria Available: http://www.R-project.org/. Accessed 20 January 2015.

[18] Revell LJ. phytools: an R package for phylogenetic comparative biology (and other things). Methods Ecol Evol. 2012; 3.2: 217–223.

[19] Csősz S, Radchenko A, Schulz A. Taxonomic revision of the Palaearctic *Tetramorium chefketi* species complex (Hymenoptera: Formicidae). Zootaxa 2007; 1405: 1–38.

[20] Lessells CM, Boag PT. Unrepeatable repeatabilities, a common mistake. Auk 1987; 104: 116–121.

[21] Yoder MJ, Mikó I, Seltmann KC, Bertone MA, Deans AR. A gross anatomy ontology for Hymenoptera. PloS ONE 2010; 5: e15991 10.1371/journal.pone.0015991 21209921 PMC3012123

[22] Seltmann KC, Yoder MJ, Mikó I, Forshage M, Bertone MA, Agosti D et al A hymenopterists’ guide to the Hymenoptera Anatomy Ontology: utility, clarification, and future directions. J Hymenoptera Res. 2012; 27: 67–88.

[23] Mikó I, Copeland RS, Balhoff JP, Yoder MJ, Deans AR. Folding wings like a cockroach: a review of transverse wing folding ensign wasps (Hymenoptera: Evaniidae: Afrevania and Trissevania). PLoS ONE 2014; 9: e94056 10.1371/journal.pone.0094056 24787704 PMC4008374

[24] Harris RA. A glossary of surface sculpturing. California Department of Food and Agriculture, Bureau of Entomology, Occasional Papers 1979; 28: 1–31.

[25] Maechler M, Rousseeuw P, Struyf A, Hubert M, Hornik K. cluster: Cluster Analysis Basics and Extensions. 2014; R package version 1.15.3.

[26] Venables WN, Ripley BD Modern Applied Statistics with S. 2002; (4th ed.) New York: Springer. ISBN 0-387-95457-0.

[27] Nilsen G, Lingjaerde OC. clusterGenomics: Identifying clusters in genomics data by recursive partitioning. 2013; R package version 1.0. Available: http://CRAN.R-project.org/package=clusterGenomics 10.1515/sagmb-2013-0016 23942354

[28] Tibshirani R, Walther G, Hastie T () Estimating the number of clusters in a data set via the gap statistic. J R Stat Soc. 2001; 63: 411–423.

[29] Beleites C, Sergo V (2012) hyperSpec: a package to handle hyperspectral data sets in R. R package version 0.98–20120923. J. Stat. Software, in preparation(http://hyperspec.r-forge.r-project.org/index.Html).

[30] Csősz S, Fisher BL. (2016) Taxonomic revision of the Malagasy members of the *Nesomyrmex angulatus* species group using the automated morphological species delineation protocol NC-PART-clustering. PeerJ 4:e1796 10.7717/peerj.1796 26989630 PMC4793320

[31] Brown JL, Cameron A, Yoder AD, Vences M. A necessarily complex model to explain the biogeography of the amphibians and reptiles of Madagascar. Nature Commun. 2014; 5: p. e5046 10.1038/ncomms6046 25297804

[32] Vences M, Wollenberg KC, Vieites DR, Lees DC () Madagascar as a model region of species diversification. Trends Ecol Evol. 2009; 24: 456–465. 10.1016/j.tree.2009.03.011 19500874

[33] Schatz GE. Endemism in the Malagasy tree flora *In* Biogeography of Madagascar (eds Lourenço W.R. and Goodman S.M.) pp. 1–9. Mémoires de la Société de Biogéographie, Paris; 2000.

[34] Cornet A. Essai de cartographie bioclimatique à Madagascar Notice Explicative. Paris: Editions ORSTOM; 1974.

[35] Richmond MP, Johnson S, Markow TA () Evolution of reproductive morphology among recently diverged taxa in the *Drosophila mojavensis* species cluster. Ecol Evol. 2012; 2: 397–408. 10.1002/ece3.93 22423332 PMC3298951

